# Who Reads Criticism Matters: How Selective Exposure Affects Public Backlash to Foreign Shaming

**DOI:** 10.1093/poq/nfag027

**Published:** 2026-05-08

**Authors:** Jamie J Gruffydd-Jones

**Affiliations:** Assistant Professor, School of Economics and Political Science, Keynes College, University of Kent, Canterbury, Kent, UK

## Abstract

Recent experimental studies have found that foreign shaming can be counterproductive, engendering more positive public opinion toward a target government. This paper shows that whether foreign shaming in fact leads to this kind of backlash is dependent on citizens’ exposure to the shaming. A modified participant preference trial finds that less nationalist Chinese citizens are significantly more likely to choose to read about American criticism of COVID-19 policies in China. While the criticism has no impact on those respondents who choose to read it, it significantly increases support for the Chinese government among those who choose not to. These findings demonstrate the importance of understanding which members of the public are exposed to international actions, and how campaigns to highlight these actions may make a backlash more likely.

Publicly naming and shaming other countries is a common tactic by which international actors seek to change their behavior (Hafner-Burton 2008; Falkner 2016; Kelley and Simmons 2020; Snyder 2020; Tingley and Tomz 2022). One way shaming can achieve this change is through the target country’s citizens, by reducing their support for their government’s actions and pressuring it into reforms (Keck and Sikkink 1998; Risse, Ropp, and Sikkink 1999; Davis, Murdie, and Steinmetz 2012; Tingley and Tomz 2022; Terman 2023).

And foreign shaming does have real impacts on public opinion. Some survey experiments show that when people are told about international criticism of their country, they do indeed become more likely to disapprove of their government’s behavior and call for reforms (Ausderan 2014; Koliev, Page, and Tallberg 2022; Tingley and Tomz 2022). But a body of recent experiments has found that these impacts are not always as positive as hoped. In countries from China to Israel, people told about foreign shaming instead become more supportive of their government and more likely to back its norm-violating policies (Gruffydd-Jones 2019, 2022; Terman and Gruffydd-Jones 2019; Greenhill and Reiter 2022; Terman 2023; Bassan-Nygate 2024; Abramson, Menon, and Gitlin 2025).^1^

What is not accounted for by these forced-choice experimental studies is whether members of the public would even encounter any foreign shaming. Does shaming have the same positive or counterproductive effects among the people who actually read about it? If the people who actively choose to digest news of these international actions respond in a qualitatively different way to the rest of the population, then there may be a systematic conflict between our experimental findings and these actions’ real-life effects.

In this article, I examine the impact of foreign shaming on both those citizens who choose to read about this shaming and those who choose not to, using a preregistered^2^ experiment with internet users in China. I find that in contrast to predictions, less nationalist respondents were significantly more likely to say they would choose to read a news story about “hostile” American criticism of their government’s early response to the COVID-19 outbreak. For these respondents, criticism had little effect. However, for those more nationalist citizens who did not originally choose to read about criticism, exposure evoked a backlash—but a mixed one. Reading the criticism made them significantly more positive toward their government and their country in general but had little impact on satisfaction with its performance on COVID-19.

This study shows that the impact of international shaming on domestic attitudes depends on who is exposed. In this study, shaming only evokes a backlash when it reaches those who choose not to read about it. If this finding generalizes, then many backlashes identified in the literature may not hold. The only shaming that is likely to engender a backlash is shaming that is front-page news and shared widely on social media. We would, therefore, expect leaders looking to encourage a backlash to campaign heavily on foreign criticism, pushing it widely across nationalist news outlets and social media.

## Experiments on the Impact of Shaming on Public Opinion

Every experimental study on foreign shaming uses a forced choice design—where respondents are randomly allocated news of shaming whether they wanted to read it or not—and examines the average treatment effect (ATE) across all respondents. The same is true of most experiments on the impacts of other foreign actions on public opinion, from institutional endorsements (Wallace 2019; Greenhill 2020) and electoral interventions (Tomz and Weeks 2020) to legal rulings (Chilton 2014; Cope and Crabtree 2020). But in the nonexperimental world, these pieces of news are not always salient, and the public’s knowledge of them is often limited (Brutger and Strezhnev 2022).

In the first place, this means we may be overestimating the impacts of this information, impacts that may only apply to a small proportion of the population who know about it. Experimental studies may identify the correct valence, showing that the information shifts public opinion in a particular direction in the aggregate, or that it *could* have an impact if salient enough. In this case, scholars can address the generalizability of their findings or recognize that they only apply to politically aware subgroups (Barabas and Jerit 2010; Gaines and Kuklinski 2011).

There is a potentially deeper problem, however. There is a risk of systematic conflict between experimental findings and real-life effects if those who choose to read about or seek out news react in a qualitatively different way to those who do not. If those who have positive reactions never read about it, and those who have negative reactions avidly seek it out, or vice versa, then the overall impact on the population could be very different. Take Abramson, Menon, and Gitlin’s (2025) finding that non-Israeli shaming makes Israelis less supportive of efforts to improve Palestinians’ human rights. Now suppose (hypothetically) that most Israelis did not seek out news of this kind of shaming and the small proportion who did were people who would react positively. In real life, shaming would have the opposite effect to the study’s ATEs.

To complicate matters further, how well experimental findings predict real-life effects may depend on political factors, especially in authoritarian states, where the information that reaches the public is shaped by the regime. It may choose to censor international news that would otherwise be of wide interest, and then only discovered by those who actively seek it out—or it can flood the market with an obscure foreign action that will even reach people who want to avoid it. Since the regime may systematically choose to censor or propagate depending on domestic or international politics (Gruffydd-Jones 2022; Terman 2023), if deliberate and inadvertent consumers of this information responded in qualitatively different ways, we would miss an important variation in public opinion.

Imagine a UN resolution condemning an authoritarian regime. Suppose that the ATE is positive—on average, news about the resolution reduces people’s support for their regime—but the effects vary by willingness to read about the news. People who *do not* want to read about it become less supportive of the regime, but those who *do* become more supportive. Now suppose this resolution occurs at a time of geopolitical tension, and the targeted leaders decide to use it as anti-foreign propaganda and broadcast it widely. Everyone is exposed to the news and we see something approaching the ATE, an increase in anti-government dissent. But suppose instead that the resolution occurs amid domestic unrest, and with the government censoring sensitive news, only those who really want to find out about the resolution are exposed, which instead leads to an increase in pro-regime sentiment.

Identifying who chooses to read about a piece of political information, and how they respond, is crucial for understanding the conditions under which that information shifts public opinion—and may be especially important in authoritarian states. Foreign shaming has features that make this especially relevant. Not only do its impacts vary dramatically across contexts, but shaming has an intrinsically emotional character (Snyder 2020), which, unlike more neutral endorsements or legal pronouncements, may lead people to systematically choose to seek it out or avoid it in ways that also influence their responses.

## Who Chooses to Read About Foreign Shaming of Their Country?

The literature on selective exposure shows that people choose information that plays into their preconceptions (Stroud 2017). They may do so to reduce cognitive dissonance (Festinger 1957, 1964); because it requires less effort to process like-minded views (Ziemke 1980); or because they believe that those views must be more trustworthy (Metzger, Hartsell, and Flanagin 2020). In the United States, there is evidence that people select sources (Iyengar and Hahn 2009; Garrett and Stroud 2014) and stories (Hart et al. 2009; Knobloch-Westerwick and Meng 2009) that accord with their own political views. In China, Huang and Yeh (2019) find that the less pro-regime and more pro-West Chinese citizens are the most likely to choose to read positive news about foreign countries.

The argument is that people want news about countries that plays into their existing views—if they feel positively about the West and negatively about China, they choose news that is consistent with those views. We may see something similar for criticism. Individuals who are more nationalistic, for whom pride in their nation is especially important to their identity, may prefer to encounter information that bolsters that pride and avoid information that challenges it. People with lower attachment to their nation may not face the same cognitive dissonance from criticism and will be able to choose to read about it on its own terms.

Together, this gives us the following hypothesis:

H1: Citizens with higher nationalist sentiment will be less likely to choose to read about foreign criticism of their government.

The puzzle is that foreign criticism is a regular feature in nationalist-leaning newspapers (Gruffydd-Jones 2022). In China, the state-owned commercial tabloid *Global Times*—well known for its hawkishness (Fish 2017)—frequently publishes foreign criticism of China, writing over one hundred stories about American criticism of China’s human rights across the last twenty years (Gruffydd-Jones 2022). In the UK, right-wing nationalist tabloids like the *Daily Mail* have reveled in news of outside condemnation of Conservative government policies.^3^

This may be simply because it is what readers want. “Nationalist” citizens—those for whom their national identity is especially important—are generally more attuned to their country’s relative status, something highlighted by foreign criticism.^4^ Emotions may also play a role. Critical information about one’s social group can spark anger and defensiveness (Nauroth et al. 2015; Thürmer and McCrea 2018; Ditrich et al. 2022). There is evidence that people deliberately expose themselves to information that provokes their anger (Tamir and Ford 2012) and bolsters their mood (Berkowitz and Harmon-Jones 2004; Chester and DeWall 2017), especially if it challenges their preconceptions or identities. Indeed, people choose to read disconfirming views on controversial issues (Jang 2014; Bakshy, Messing, and Adamic 2015) and even search for negative news about their preferred political candidates (Meffert et al. 2006). This may allow them to develop more effective counterarguments on issues they are passionate about (Meffert et al. 2006) or give them “an opportunity to be antagonistic toward someone who thinks differently” (Young et al. 2011, 12). People who are most likely to respond in this way are likely to be those who care deeply about their nation’s performance and prestige.

Together, this gives us H2:

H2: Citizens with higher nationalist sentiment will be more likely to choose to read about foreign criticism of their government.^5^

### Nationalism, Selective Exposure to Criticism, and Backlash

The International Relations literature has two contrasting stories for how foreign shaming may lead a target public to influence their state’s behavior. On one side, some authors have argued that it will encourage publics to push their state into “compliance” with the shaming (Keck and Sikkink 1998; Risse, Ropp, and Sikkink 1999; Davis, Murdie, and Steinmetz 2012; Koliev, Page, and Tallberg 2022; Tingley and Tomz 2022). Terman and Gruffydd-Jones (2019) break this compliance down into three distinct “pathways” (table 1).

**Table 1.** Pathways between foreign shaming, public opinion, and target state compliance/backlash. Bold indicates compliance; italics indicates backlash.

Pathway	Shaming impacts on public opinion		Public opinion impacts on policy
1. Issue beliefs	**More**/*less* negative about government performance on the shamed issue	⟶	**More**/*fewer* government policy changes on the shamed issue in line with shaming
2. Country-related beliefs	**More**/*less* negative about government in general	⟶	**More**/*fewer* changes in wider government policies in line with shaming
3. Issue activism	**More**/*less* supportive of wider activism over the shamed issue	⟶	**More**/*less* government responsiveness to activism on shamed issue and more widely

The simplest path to compliance is a Bayesian one (pathway 1): Foreign shaming informs the public about their government’s poor performance on the issue being shamed and that outside actors see it as worthy of disapproval. Individuals negatively update their views about the specific criticized policies, which may lead to targeted policy changes if governments are responsive to public opinion (Davis, Murdie, and Steinmetz 2012; Koliev, Page, and Tallberg 2022; Tingley and Tomz 2022).

For many policies, people’s views are settled and unlikely to be shifted by foreign criticism (Hendrix and Wong 2013). But even then, shaming may make them see other local and international efforts to put pressure on the government as more legitimate (Simmons 2009). In this case, foreign shaming may not change how people think about their government’s policies but make them support more scrutiny of them, scrutiny that may itself lead to compliance (pathway 3).

Finally, shaming may increase people’s broader discontent with their government or country (pathway 2). As Finnemore and Sikkink (1998, 904) say, “citizens make judgments about whether their government is better than alternatives…by seeing what other people and countries say about their country.” Citizens may hold firm views about the criticized issue or not care about it at all, but just hearing other states’ disapproval may lead them to negatively update their views about their government and country, or to vocalize their concerns about other issues. In turn, they may vocally or electorally express their discontent toward these noncompliant governments, potentially leading to wider policy changes.

Other authors have drawn on the social psychological literature to argue that foreign shaming may, in fact, reduce the likelihood that a target state’s citizens will push their government into change (Snyder 2020; Gruffydd-Jones 2022; Terman 2023). This approach focusses on the challenge that foreign shaming poses to citizens’ attachment to their nation. People’s group identities—often including their national identities—are a big part of who they are, and so when they feel that their nation is being stigmatized, many feel personally attacked (Tajfel and Turner 1986; Steele 1988; Rubin and Hewstone 1998; Sherman and Kim 2005). Rather than seeking to accurately update their views in response to any new information, they are driven by the directional goal of protecting their identity against the attack (Druckman and McGrath 2019).

This defensiveness may lead to a “backlash” on each of the shaming pathways. First, citizens may vehemently express their disagreement with the shaming itself (Nauroth et al. 2015; Trevors et al. 2016) or think through ways in which it is wrong (Redlawsk 2002; Meffert et al. 2006; De Hoog 2013). By developing or expressing these counterarguments, they may develop more positive views about the criticized policies (Greenhill and Reiter 2022; Gruffydd-Jones 2022; Terman 2023; Bassan-Nygate 2024; Abramson, Menon, and Gitlin 2025). Second, even if criticism does not shift people’s well-established views about policies, it may make them lash out against the criticizer (Hornsey and Imani 2004; Grossman et al. 2018; Thurmer and McCrae 2018; Gruffydd-Jones 2022). Citizens do not change their views about the issue but become more hostile to any efforts to criticize their country, making follow-up advocacy more difficult.

Finally, even if people do not change their views about the issue or other actors, in the face of criticism that threatens their positive group identity, they may instead rally around that group and assert its positive characteristics (Ellemers, Spears, and Doosje 2002; Major and O’Brien 2005; Branscombe et al. 2002). There is evidence that in the face of foreign criticism people become more outspokenly positive toward their leaders (Snyder 2020; Terman 2023), and some leaders have openly referenced foreign criticism in their bids for reelection (Terman 2023).

One crucial difference between backlash and compliance is whether an individual feels the need to defend their nation against the shaming. This may come down to the identity or perceived hostility of the critic (Hornsey and Imani 2004; Snyder 2020; Gruffydd-Jones 2022; Terman 2023), but also the individual’s attachment to their nation. In the social identity theory and motivated reasoning literatures, the more important people’s group identity is to their self-esteem, the more they will be concerned about defending it (Tajfel and Turner 1979; Branscombe and Wann 1994; Doosje, Ellemers, and Spears 1995; Ellemers, Spears, and Doosje 2002; Major and O’Brien 2005; De Hoog 2013; Herrmann 2017). The more attached people feel to their national identity and the more their self-worth is tied to that of their nation, the more they will feel personally attacked by criticism.

Highly attached individuals should be more likely to reject accuracy motivations and seek to fight back. They should be more likely to develop counterarguments over the criticized issue, reject further scrutiny from other actors, and rally around their country and its leadership. The naming and shaming literature has shown that any backlash is indeed more likely among more nationalist citizens (Gruffydd-Jones 2019; Spektor, Mignozzetti, and Fasolin 2022; Bassan-Nygate 2024), and that any backlash generally comes in places where governments have explicitly framed foreign pressure in defensive nationalist terms (Gruffydd-Jones 2022; Terman 2023; Bassan-Nygate 2024; Abramson, Menon, and Gitlin 2025).

### Hypotheses

If people’s attachments to their nation are indeed critical to their decision to read about foreign shaming (**H1** and **H2**), then those who want to read about shaming should respond in a qualitatively different way from those who do not want to.

If H1 is correct, a backlash should be most likely among the more nationalist individuals who do not want to read the criticism. They deliberately avoid information that leads to cognitive dissonance, but on encountering it, seek to alleviate the attacks on their self-esteem. Those who do choose to read it have less motivation to defend their nation against the critical messaging, and update their views in a Bayesian manner.

H1b: If H1 is correct, then foreign criticism will lead to more backlash among citizens who choose not to read it, but more compliance among those who choose to.

If H2 is correct, then we should be most likely to see a backlash among the more nationalist individuals who want to read the criticism. They are attuned to and emotionally stimulated by criticism of their nation and respond aggressively. Those less nationalist individuals who do not want to read the criticism update their views in a Bayesian manner when they do encounter it.

In these hypotheses, I do not ex ante distinguish between the different pathways to compliance or backlash. While they are conceptually distinct and we may see backlash on one pathway but not another, depending on the type of shaming, nationalism—and reading choice—should theoretically make any backlash more likely regardless of the pathway.

H2b: If H2 is correct, then foreign criticism will lead to more backlash among those who choose to read it, but more compliance among citizens who choose not to.

### Experimental Research Design

All experimental studies of the impacts of international “tools” on the public—from international law to shaming—use a simple forced-choice design. In this design, a whole survey sample is randomly exposed to information about the tool, whether or not the respondents would have chosen to read about it under normal circumstances. Our goal is to break down whether these tools have heterogeneous impacts on respondents who would choose to read about it versus those who would not.

**Figure 1.**
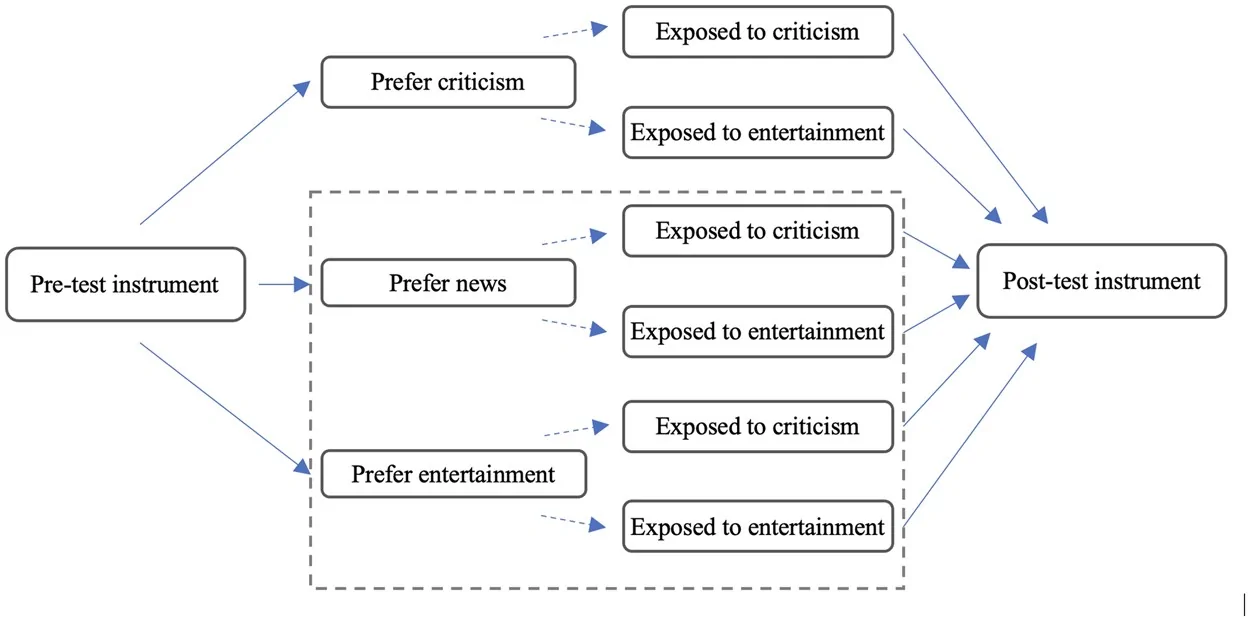
Design of the adapted participant preference trial used in this experiment.

To do so, I used an adapted participant preference trial (PPT) design, shown in figure 1. PPT designs originated in medicine^6^ and have increasingly been employed in (mainly US-based) political science studies to study the impacts of exposure to partisan media (Levendusky 2013; Arceneaux and Johnson 2015; Huang and Yeh 2017; de Benedictis-Kessner et al. 2019). The design is broadly the same: Participants are asked to first indicate their preference for networks, programs, or stories, and are then randomly assigned those stimuli. Experimenters can then explore the impacts of exposure to media content on both those who chose it and those who did not.

I examine the impact of American criticism of the Chinese government’s initial COVID-19 response. Despite the global mudslinging surrounding countries’ COVID-19 policies, no studies have analyzed its impacts. China’s response is a crucial case study. Not only did it face widespread international criticism, but the country’s biggest wave of public protests in a decade (soon after this study was carried out) came in response to COVID-19 lockdowns and were a major factor in their removal (Thornton 2023). As such, this is a case where both domestic public opinion and international criticism had potentially important impacts in influencing eventual policy decisions, but also a case that saw a visible burst in nationalist sentiment and rhetoric (Zhang 2020; Yang and Chen 2021; Zhang and Xu 2023).

Participants were recruited through a crowdsourcing company in June 2021 and directed to take the survey anonymously on Qualtrics, with 1,163 completing the survey. They were drawn from all demographics, but the sample more closely represents China’s social media users—more well educated, younger, and urban. After demographic and attitudinal questions,^7^ participants were asked to choose one of three articles that they would like to read.^8^ All were adapted from real Chinese newspaper headlines in April 2021, published just before the survey was carried out. The first “criticism” article was headlined “The U.S. condemns China’s COVID-19 response.” The second “news” article was entitled “Guangzhou’s foreign students receive the COVID-19 vaccine.” This was included as a generic description of China’s COVID-19 response, mentioning foreign actors, but without any positive or negative implications. The goal was to ensure that respondents were not picking the criticism item due to an interest in news about COVID-19, with the primary difference between the two the critical dimension of the first article. One unavoidable consequence of this design is that all control and treatment respondents see a critical headline. While the headline was deliberately free of detail, this does mean that the treatment effects should most appropriately be read as indicating the difference between reading a full account of criticism and only seeing a headline.^9^

Respondents were also given the choice of a third “entertainment” headline, entitled “Monster movies are losing their appeal.” This headline was included for two reasons. First, since many people pay no attention to political news, they may prefer to read neither of the news stories (Arceneaux and Johnson 2013). Second, including a third option allows us to employ it as a kind of placebo—to examine whether those choosing to read about criticism are doing so because they are interested in that story, or just because they do not wish to read about the other story on foreign students.

After answering about their reading preferences, respondents were randomly allocated into either a “criticism” block or an “entertainment” block. Those in the “entertainment” control block were provided with the text of the entertainment story, a story that was unrelated to COVID-19 or the United States (taken from the same real article in April 2021). Those in the “criticism” treatment block were given a news article—again adapted from a real article in April 2021—but stripped of names and the government’s response. The treatment text (translated from Chinese) read:

According to multiple U.S. media reports, on May 14 the U.S. once again blamed China for the COVID-19 epidemic at a press conference. At this press conference, the U.S. Secretary of State harshly criticized China. He claimed that China not only concealed and failed to report the epidemic at the beginning, but also did not take any necessary measures against the epidemic, which led to the global spread of the COVID-19 virus. He said: “China should be held responsible for the COVID-19 epidemic.”

This prompt reflects American criticism of China after 2020, targeting the country’s failures at the start of the pandemic, but is also designed to be as generic as possible. It mirrors the aggressive, percussive condemnation from a hostile geopolitical opponent on a topic well known by Chinese citizens—precisely the kind of criticism most likely to evoke a backlash (Gruffydd-Jones 2022). Throughout the pandemic, American criticism like this was repeatedly highlighted in Chinese state media.^10^

I then measure the Average Conditional Treatment Effect (ACTE), the average effects of exposure to the shaming story relative to the entertainment story, among the subgroup who stated that they would choose to read about shaming, and the subgroup who stated they would choose to read about something else. One issue with this “traditional” PPT design is that regardless of their stated preference, participants are still randomly exposed to a story—and so we do not necessarily know how they compare to those who would have genuinely chosen that story. To address this, some scholars compare respondents in the “randomized” arm to respondents in a “choice” arm who freely choose their story (de Benedictis-Kessner et al. 2019; Knox et al. 2019). However, in this study, we are primarily interested in understanding how reading preferences influence the effects of exposure to shaming—whether those who would choose to read about shaming differ in their responses to those who would not. The traditional PPT design that incorporates preferences prior to randomization is sufficient to address this question. For this study, adding an extra free-choice arm adds an extra level of complexity and survey cost that would not add much to this objective. And indeed, even without an explicit free-choice arm, we are still able to compare the responses of the subgroup who were given the story that they chose with those who were given a different story.^11^

The primary benefit of adding a separate free-choice arm is that it helps to address the problem of ignorability: that people’s stated preferences may not match their actual choice of story (Knox et al. 2019). I address this concern by asking participants directly for the story they would like to read, rather than for a more general media preference.^12^ Since this means that some participants will be given different news stories to their preferred choice, I inform them that researchers were interested in their response to news content that was different to their choice.^13^ While this design minimizes ignorability concerns, it may introduce issues around resentful demoralization, which I address below.

Respondents were asked to write their thoughts about the article. They were then given three sets of posttreatment questions, to reflect the separate pathways through which shaming may influence public opinion to lead to compliance or backlash (Terman and Gruffydd-Jones 2019, see table 2). While the study aims to understand the mechanisms behind backlash or compliance on each pathway, their distinct logics means that it is important to treat these pathways separately.^14^ One set directly tested the criticized issue (pathway 1), asking respondents to evaluate the local or central government’s response to the COVID-19 outbreak. A second examined general country-related beliefs (pathway 2), asking respondents to rate their overall satisfaction with their local and central government, their nation, and its political system. The third examined support for international and domestic activism (pathway 3), asking respondents whether they would support inquiries into the COVID-19 response.

**Table 2.** Outcome variables, by distinct shaming pathways. Full texts included in the Supplementary Material.

Pathway	Variable
1. Issue beliefs	How well central government dealt with COVID-19 outbreak How well Wuhan government dealt with COVID-19 outbreak
2. Country-related beliefs	Satisfaction with central government Satisfaction with local government Belief that China is the best country in the world Belief that China is a democratic country
3. Issue activism	Support for domestic media inquiry into (Chinese) authorities’ initial handling of COVID-19 Support for civil Chinese inquiry Support for United Nations inquiry

## Results

### Selective Exposure to Criticism

Just over a third of the sample (397 respondents) stated that they would choose to read about American criticism, with 53 percent choosing the news story and 13 percent the entertainment story. In contrast to my pre-analysis prediction, but providing support for H1, more nationalist Chinese citizens were *less* willing to read about American criticism of their government. As table 3 shows, nationalist sentiment was by far the strongest predictor of reading behavior, with those choosing to read about criticism 12 percentage points more likely to report above-average nationalism. Apart from social media use, no other variable showed a significant impact on reading preference.^15^ These results may not just be a case of less nationalist citizens choosing to read about criticism, but merely a consequence of more nationalist respondents choosing to read the news story about vaccines. But if this was driving the response to criticism, we would also expect to see lower nationalism among those who chose the entertainment story—but this was not the case.^16^

**Table 3.** Balance table on preference for reading about foreign criticism (versus other two reading options). For ease of interpretation and comparison, all nondichotomous variables are broken down into dichotomous variables (above/below mean value). See Supplementary Material figure A1 for results from logistic regressions. Difference reported is a two-way t-test. Standard deviations in parentheses and *p*-values for the difference in italics.

	Did not choose criticism	Chose criticism	Difference
High nationalism	0.736	0.615	−0.122
	(0.441)	(0.487)	*p* = 0.000018
Like US government	0.444	0.436	−0.008
	(0.497)	(0.496)	*p* = 0.792
Like US people	0.517	0.554	0.037
	(0.500)	(0.498)	*p* = 0.229
Liberal political ideology	0.428	0.474	0.045
	(0.495)	(0.500)	*p* = 0.140
High news from social media	0.807	0.846	0.040
	(0.395)	(0.361)	*p* = 0.087
High news frequency	0.345	0.322	−0.022
	(0.476)	(0.468)	*p* = 0.445
High news interest	0.540	0.557	0.016
	(0.499)	(0.497)	*p* = 0.599
Good knowledge of US politics	0.183	0.186	0.004
	(0.387)	(0.390)	*p* = 0.880
Female	0.594	0.559	−0.035
	(0.491)	(0.497)	*p* = 0.256
City province	0.311	0.305	−0.006
	(0.463)	(0.461)	*p* = 0.836
Over 35	0.351	0.388	0.037
	(0.478)	(0.488)	*p* = 0.220
Han ethnicity	0.975	0.977	0.002
	(0.156)	(0.149)	*p* = 0.814
Undergraduate degree	0.799	0.821	0.022
	(0.401)	(0.384)	*p* = 0.357
Rural Hukou	0.846	0.878	0.033
	(0.362)	(0.328)	*p* = 0.123
Observations	766	397	1,163

*Note:* Standard deviations in parentheses.

### Impact of Selective Exposure to Criticism

To examine the impact of selective exposure, I carried out adjusted OLS with covariates for each of the outcomes above.^17^ To address concerns around multiple outcomes, I follow the pre-analysis plan and combine posttreatment questions along the pathways through which backlash or compliance might occur: toward the issue itself; toward the regime/country in general; and toward other activism. Multiple hypothesis testing does not change the results (Supplementary Material section 4, table A7).

**Figure 2.**
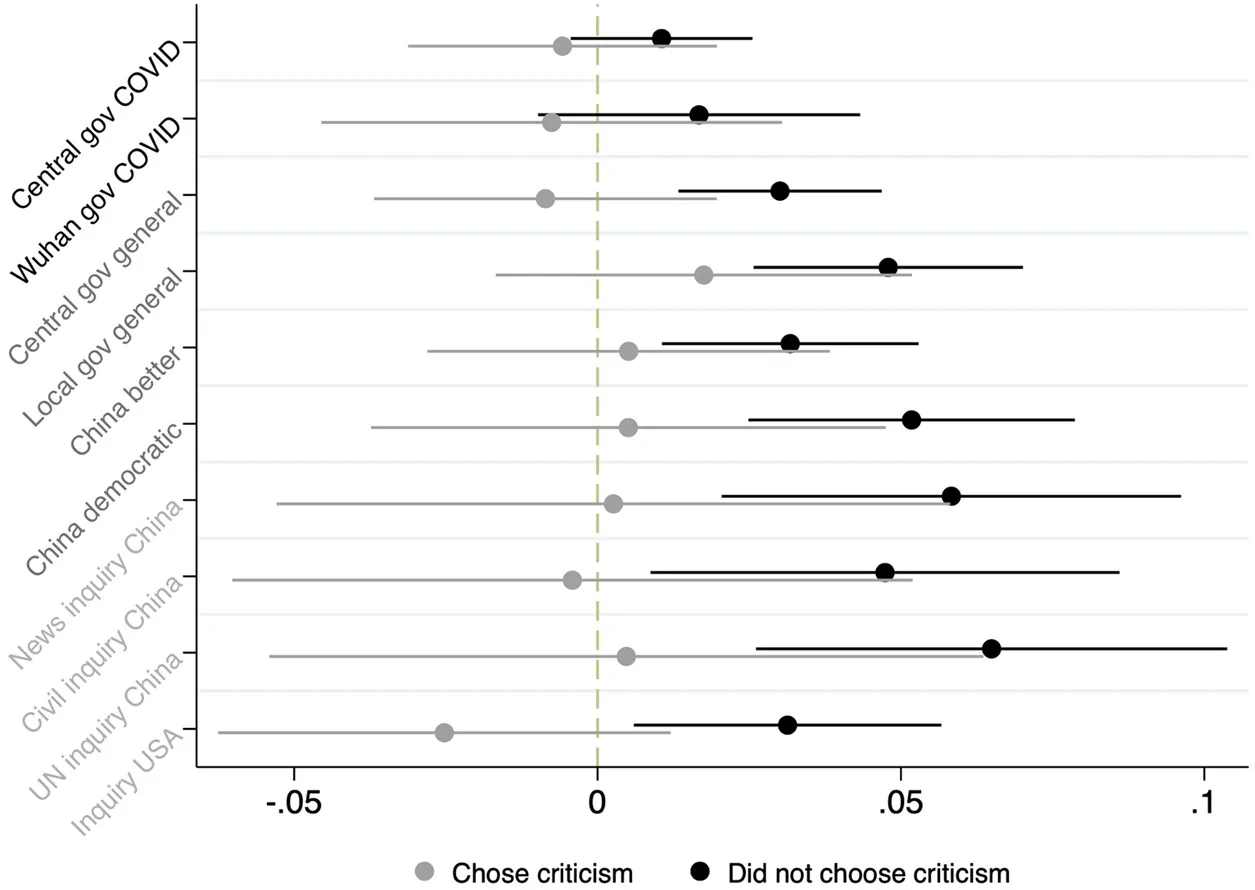
Average conditional treatment effects of criticism by reading preference. Outcomes are standardized from 0 to 1, using adjusted OLS with covariates, where higher value equates to more support for the variable. Horizontal lines represent 90 percent confidence intervals. Support for independent inquiry into the United States’ original COVID-19 response included for comparison purposes.

**Figure 3.**
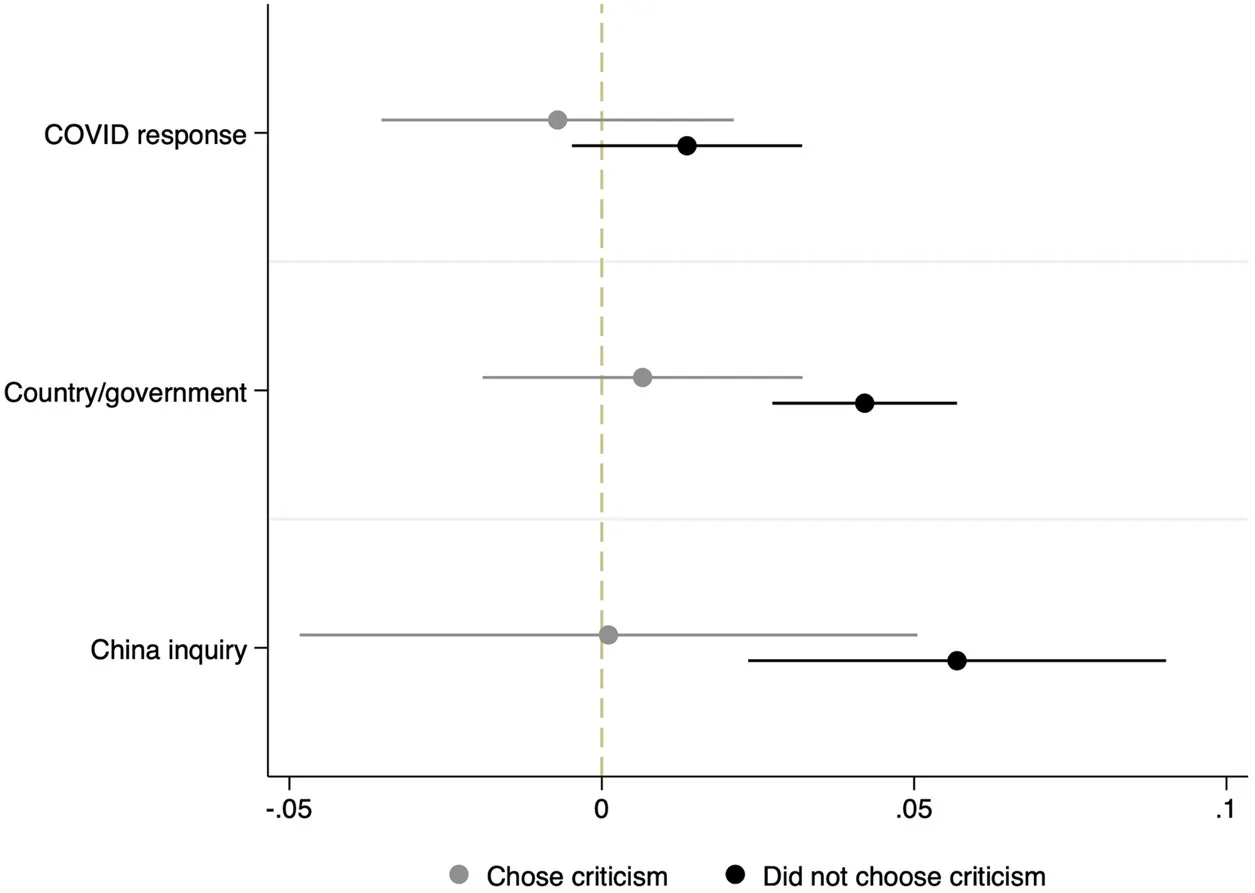
Average conditional treatment effects of criticism by reading preference, questions combined along compliance-backlash pathways. Note that higher values for COVID response and country/government indicate backlash, but for China inquiry indicates compliance. Support for inquiry into the US response not included. Outcomes standardized between 0 and 1, adjusted OLS with covariates, where higher value equates to more support for the variable. 90 percent confidence intervals.

On average, American criticism of China’s COVID-19 response led to a backlash among Chinese respondents. But the impact of criticism was heavily dependent on respondents’ reading preferences as well as the route of the backlash. Figure 2 shows the ACTEs by outcome question, and figure 3 shows the results combined along the compliance/backlash pathways.

Criticism only showed a backlash among those who stated that they would choose not to read it. Respondents who initially chose to avoid criticism became significantly more positive toward their government and country after reading it. Criticism increased satisfaction with central and local governments by 3 and 4.8 percentage points, respectively, and made people 3.2 and 5.2 points more likely to believe their country was the best in the world and democratic.^18^ However, criticism had only minimal impacts on attitudes toward COVID-19 policies, regardless of reading choices. It also made nonreaders significantly *more* willing to support independent inquiries into their government’s initial response to COVID-19, by between 5 and 6 points.^19^ On the other hand, American criticism had no impact on those who stated that they would choose to read about it, with effect sizes almost all less than one point.^20^ As figure 3 indicates, criticism was significantly more likely to lead to increased support for the regime^21^ and for an independent inquiry^22^ among those who chose not to read about criticism compared to those who chose to read it.

Preferring to read a neutral over a critical story does not necessarily mean people do not want to read the critical story. To examine this, I also asked respondents whether they would want to read any of the other articles, if they had a choice. Comparing those respondents who indicated they would like to read the criticism *at any point* with those who said they would not gives us similar results to the above (with the notable exception of the independent inquiries: Both groups showed more support for inquiries after being told about criticism; see Supplementary Material section 4, figure A15).

The results may also reflect pretreatment effects (Gaines, Kuklinski, and Quirk 2007), whereby the “readers” had also previously read about real-life criticism of China’s COVID-19 response, and therefore had already factored any backlash to criticism into their views. This concern is diminished by the sheer prominence of the issue itself, with COVID-19-related criticism widespread on all Chinese media platforms, including nationalist-leaning ones.^23^ Respondents who said they regularly read or were interested in the news were no more likely to choose to read the criticism—suggesting that previous exposure is unlikely to explain these results.

Importantly, these results also do not appear to be due to “resentful demoralization” (Bradley 1993), whereby those not given their chosen piece of news become frustrated at the criticism. First, those who chose criticism but received the entertainment story, who should also be resentful at not receiving their choice, did not show any backlash. Second, all respondents who chose the neutral news headline subsequently read stories they did not choose (either entertainment or criticism). Even if we limit our analysis only to this neutral news group, we still find that criticism caused a backlash, whereas entertainment did not (Supplementary Material section 4, figure A12).

## Discussion

This study finds that while nationalist Chinese citizens avoid criticism of their nation, inadvertent exposure bolsters their (already highly) positive views about their government. Criticism had no impact, however, on those who chose to read about it. A plausible explanation is that the higher nationalism of nonreaders predisposes them to react defensively. The results also support the existence of three distinct routes through which shaming can influence public opinion (Terman and Gruffydd-Jones 2019). In this study, shaming increased generic government support, but not support for its performance on the criticized issue. This is plausibly because at the time of the study, people’s views on COVID-19 had been cemented by their own experiences over the past year and were therefore less vulnerable to criticism from external voices. Criticism may have a stronger effect on issues on which people have not yet made up their mind. It does show, however, that even when shaming has a minimal impact on the policy itself, it may nonetheless rally people around their leaders and thus strengthen political support.

A more surprising finding was that shaming made nonreaders more supportive of inquiries into authorities’ handling of COVID-19. On one hand, this may be because reading criticism about COVID-19 increased people’s concerns about the virus, making them more resolved to uncover its origins. Indeed, the criticism also made nonreaders more supportive of an inquiry into the US COVID-19 response. On the other hand, the fact that this effect only arose among those who initially chose not to read the criticism (and among those higher in nationalism and more negative towards the United States^24^) suggests that the reaction may be a defensive one. An inquiry might be a way for citizens hurt by criticism to prove that their government dealt with the virus properly.

While this study sought to move beyond the strict forced-choice experiments that characterize this field, there are of course limitations. The control group also read a headline about criticism. While this means that the study will, if anything, underestimate any backlash from nonreaders, it will also overestimate the likelihood of null results in the readers. Given that the headlines were given to both those who chose to read the criticism and those who chose not to, however, they should not exaggerate the difference in responses between the two.^25^

The experiment did not include a distinct “free-choice” design, so we cannot fully compare the findings with what would happen if respondents had complete freedom to choose what they wanted to read.^26^ Respondents were also only provided with three choices, which hardly reflects how they would deal with information in their normal lives. We cannot account for those citizens who might not have chosen any of these stories, or who might actively seek out criticism not available in everyday news.

The online sample is also more representative of social media users than the overall population: younger, more educated, and more urban. While studies with crowdsourced Chinese samples are generally consistent with population-based surveys (Li, Shi, and Zhu 2018), this does mean that our point estimates for reading likelihood or government support will not exactly match the population. Our main hypotheses are unlikely to be affected, however. Demographic characteristics did not affect reading likelihood (table 3), nor did they affect the impacts of criticism on government support (Supplementary Material section 5).

**Figure 4.**
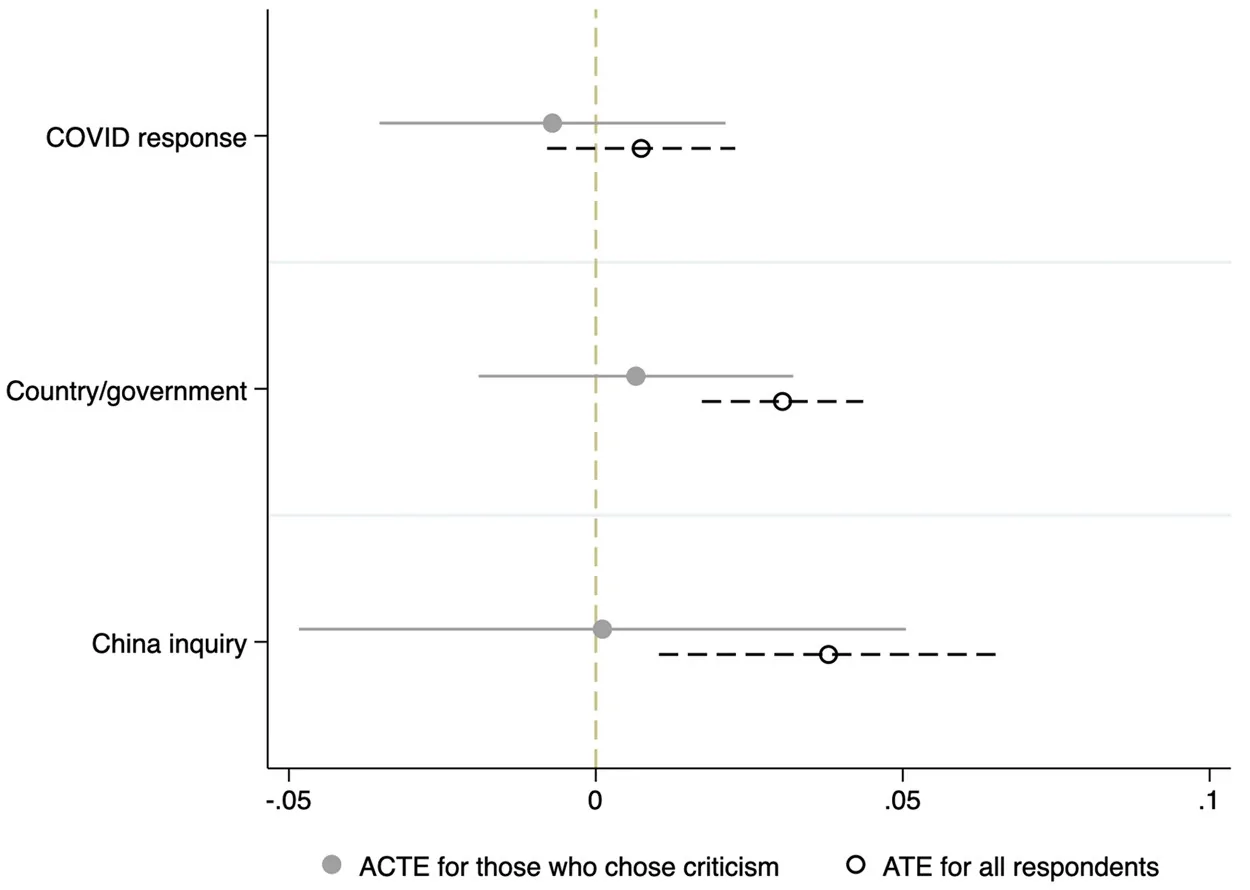
Average treatment effects for all respondents compared to average conditional treatment effect for those who chose to read criticism, questions combined along compliance-backlash pathways. Support for inquiry into the US response not included. Outcomes standardized between 0 and 1, adjusted OLS with covariates, where higher value equates to more support for the variable. 90 percent confidence intervals.

The study nonetheless demonstrates that research into the impact of international shaming on public opinion needs to consider who knows about it. As Gaines and Kuklinski (2011, 735) say: “if self-selection of treatment…is probable, then there is good reason to estimate the treatment effect separately for those who do, as a general rule, experience the treatment and those who do not.” In this case, the minority who chose to read about the treatment had a very different reaction to those who chose to avoid it. As figure 4 shows, results from the full model are notably different to the results for only those who chose to read about shaming. The backlashes identified through average treatment effects in forced-choice experimental studies may, therefore, overestimate shaming’s true impact. We should interpret these studies as showing that shaming may well lead to a backlash—but only if it is high-profile or salient enough to reach those who were inadvertently exposed to it. Since most shaming reaches only the small proportion of people who seek it out, the real-life effects may, in fact, be nonexistent.

This also implies that the shaming most likely to cause a backlash is the shaming that reaches people who would not normally choose to read about it. In China, some foreign criticism needs to be actively sought out. Other criticism is taken up enthusiastically by propaganda officials and becomes most-read posts on social media. While spreading news that international actors have been shaming the government over its failures risks telling previously unaware people about those failures (Fang 2021), this study shows that this may be the point—it is those people who would not otherwise read about criticism who would be the participants in a nationalist backlash. To engineer such a backlash, governments need to highlight foreign criticism as widely as they can. This helps answer the puzzle posed in hypothesis 2: Nationalist regime-supporting outlets do not broadcast criticism because that is what their readers are interested in—but because it serves the regime’s propaganda purposes.

These findings may not extend to all forms of international public diplomacy. The emotional and psychological challenge of foreign shaming makes it especially likely to provoke selective exposure. Other “drier” tools, from endorsements to legal obligations, may not attract or repel readers in such a systematic way. The more that shaming evokes avoidance and defensiveness, the more it will lead to this kind of selective backlash, implying that we will be more likely to see these kinds of findings on criticism that is seen as particularly hostile or that targets especially nationalistic topics.

Human rights are likely to be one such topic. Regimes like China or Israel that are regularly targeted by the international community over human rights often explicitly “weaponize” this criticism in a battle of national pride (Gruffydd-Jones 2019; Abramson, Menon, and Gitlin 2025). We may not see the same kind of selective exposure effects in countries where human rights shaming has not been weaponized in this way, or on other topics like air pollution or corruption that have not (yet) been tied to the national identity (Gruffydd-Jones 2022).^27^

For states or NGOs designing a shaming campaign, the study provides some nuance to previous research suggesting that their efforts might provoke a backlash. It implies that any backlash may not be as widespread as experimental studies suggest, but will depend on the reach of the criticism. Low-profile criticism that only reaches those who seek it out will have little negative effect, while high-profile criticism that reaches those people who did not seek it out will lead to a backlash. Counterintuitively, then, this implies that if there is a risk of a backlash, campaigners will have an interest in *not* publicizing their criticism widely in the target country, and keep their shaming efforts away from pro-regime media, such that it only reaches those citizens who seek it out.

## Supplementary Material

nfag027_Supplementary_Data

## Data Availability

Replication data and documentation are available at https://dataverse.harvard.edu/dataset.xhtml?persistentId=doi:10.7910/DVN/IQV3PE.

## Footnotes

1 See Supplementary Material section 7 for a fuller literature review.

2
https://osf.io/cq7wh/overview?view_only=2c4d2d775b5d43fd8700281698d2a468.

3 For example, Martin (2021) and Wright (2021) from April 2021.

4 For example, Mutz and Kim (2017).

5 This was the primary hypothesis in the pre-analysis plan.

6 See Wasmann et al. (2019).

7 Full survey questions in Supplementary Material section 1. The randomization procedure was generally successful (Supplementary Material section 2, table A2).

8 While choosing which one they “would like to read” is not necessarily the same as picking one to read, a post hoc experiment in the United States found no difference in responses to the two wordings. See Supplementary Material section 3, note 2. References to “choice” in this article refer to this preference to choose.

9 See Supplementary Material section 6.

10 See, for example, *People’s Daily* (2020a); *People’s Daily* (2020b); *People’s Daily* (2020c).

11 See Supplementary Material figure A13.

12 See footnote 8 and Supplementary Material section 3, note 2.

13 Participants were told the true goal of the study in the debrief.

14 These distinct routes are set out in the pre-analysis plan.

15 See Supplementary Material section 3, note 1, for a discussion.

16 See Supplementary Material figures A2/A3.

17 Unadjusted estimates and non-OLS regressions in Supplementary Material section 4.

18 All *p *< 0.005, except for the “best country” measure (*p *=0.01). For the combined measure, *p *< 0.001. Multiple hypothesis testing using Anderson (2008) gives q-values of similar significance (Supplementary Material table A7).

19 For the combined measure, *p *< 0.0001.

20 The exception was on local government, with (nonsignificant) effect size of 2.9 p.p.

21
*p *= 0.031 for the interaction, Supplementary Material table A6.

22
*p *= 0.092 for the interaction.

23 In a pilot study (two months earlier), 174 of 210 respondents said they had heard about foreign criticism of COVID-19 policies in China, with no difference by nationalism.

24
Supplementary Material figures A16 and A21.

25 See Supplementary Material section 6 for a fuller discussion.

26 See Supplementary Material figure A13 for an approximation of this.

27 See Supplementary Material section 7 for a fuller discussion.

## Appendix A. AAPOR-Required Disclosure Elements

**First Data Source** 

Survey data collected by author through online sources.

**Data Collection Strategy** 

Data was collected via a survey.

**Research Sponsor and Conductor** 

Research sponsored by the University of Kent’s School of Humanities and Social Science. Research was conducted by the author alone, with the assistance of an anonymized market research company. It has become best practice in social science survey experiments to anonymize the names of local market research platforms in China (see Lü 2015 for a fuller discussion of these ethical issues and Huang 2025 for a recent example of this same nondisclosure on a similar topic).

**Measurement Tools/Instruments** 

Questionnaire with survey questions, instruments, and instructions included in full in the Supplementary Material.

**Population Under Study** 

Chinese internet users (aged 18 and over) who were members of the online panel maintained by (anonymized) market research company in June 2021. No other social, geographical, or demographic restrictions.

**Methods Used to Generate and Recruit the Sample** 

Nonprobability sample through opt-in to market research panel (anonymized) with over five million members from across China. No quotas were used, but under-18s and respondents from outside the Chinese mainland (including Hong Kong and Macao) were excluded from the study. Members of the panel join with the goal of completing online surveys for possible rewards. Possible participants receive a link in their email from the market research company inviting them to take part in a survey. They can choose freely whether to enter the survey or not.

As part of the panel, participants are given incentives to participate on a points system. Those points can be pooled and later redeemed in the form of gift cards, skymiles, credit for online games, and so on. These points are offered for all surveys, depending on their length, and typically equate to a small reward. Participants usually complete a few surveys before they are able to convert them into prizes. This survey was short, so the number of points received was relatively small. There are no other incentives for participation.

**Method and Mode of Data Collection** 

Survey data collected through Qualtrics’ online platform in Simplified Chinese only.

**Dates of Data Collection** 

Data collection from June 9 to 10 2021.

**Sample Size** 

Sample size included 1,163 participants.

**Whether and How the Data Were Weighted** 

No weights used.

**How the Data Were Processed and Procedures to Ensure Data Quality** 

No attention checks. All respondents included, but respondents excluded in robustness checks if they responded in the bottom tenth percentile (see Supplementary Material figure A11). Respondents were screened by the market research panel to ensure that they were (a) not bots and (b) could not complete the study more than once. Coding of variables was done only by the author.

**Panel Description** 

As described above: The survey used a market research panel (anonymized) with over five million members from across China. Procedures for membership, participation, and attrition carried out by the company. Members of the panel join with the goal of completing online surveys for possible rewards. Possible participants receive a link in their email from the market research company inviting them to take part in a survey.

**Screening Criteria and Process** 

Screening to exclude under 18 s and respondents outside mainland China. Screening carried out by market research company, and questions included in survey (none were identified).

**Study Stimuli** 

None, except for the randomized exposure to the treatment text, described above. Full texts given in the Supplementary Material.

**Dispositions or Response or Participation Rates** 

No information on nonparticipants, as this is an opt-in survey and the survey was completed when the number of participants was full. The survey was filled within one day.

**Measurement and Model Specification** 

Replication data and documentation are available at https://dataverse.harvard.edu/dataset.xhtml?persistentId=doi:10.7910/DVN/IQV3PE. Main results used adjusted OLS with covariates. Unadjusted estimates and non-OLS regressions included in the Supplementary Material. All variables used are detailed in full in the Supplementary Material.

**General Statement Acknowledging Limitations of the Design and Data Collection** 

The data relies on a sample taken from an opt-in online panel. It is therefore nonrepresentative of the overall Chinese population. It overweights participants who are likely to join online market research panels and complete online surveys on “social attitudes” (more likely to be younger, more educated, and urban, as detailed in the Supplementary Material).
