# Supplementary material for: Who Reads Criticism Matters: How Selective Exposure Affects Public Backlash to Foreign Shaming
Source: Public Opin Q. 2026 May 8;90(SI):1144–72. doi: 10.1093/poq/nfag027 (PMC13553010; doi:10.1093/poq/nfag027)
Supplement: Supplementary file 1 [file nfag027_supplementary_data.pdf]

Who Reads Criticism Matters: How Selective Exposure Affects Public Backlash to Foreign Shaming

Jamie J. Gruffydd-Jones

University of Kent

j.j.gruffydd-jones@kent.ac.uk

Supplementary Material

TABLE OF CONTENTS

	Section	Page
I.	SURVEY (in English)	2
II.	SAMPLE & ATTRIBUTES	6
III.	READING CHOICE	7
IV.	EFFECTS OF SHAMING	12
V.	IMPACT BY PRETREATMENT CHARACTERISTICS	22
VI.	IMPACT OF HEADLINES	24
VII.	LITERATURE ON NAMING AND SHAMING	25
VIII.	PREANALYSIS PLAN	26
IX.	REFERENCES	29

I. SURVEY

The survey is below in English (Chinese on request). First, respondents were asked some background demographic questions. They were then asked about their news preferences, including their choices for reading one of three news stories, followed by questions about some social and political attitudes. Finally, they were provided with the randomised experiment described in the article, followed by the outcome questions described in the article.

A. BACKGROUND QUESTIONS

D1. What is your gender?

- <01> Male
- <02> Female
- <99> No answer

D2. In what province do you live?

D3. How old are you?

- <01> 0 - 18
- <02> 19 - 29
- <03> 30 - 39
- <04> 40 - 49
- <05> 50 - 59
- <06> 60 -
- <99> No answer

D4. What is your ethnicity?

- <01> Han
- <02> Minority
- <99> No answer

D5. What is your highest education level?

- <01> None
- <02> Primary School
- <03> Middle School
- <04> High School or Equivalent
- <05> Professional Certificate
- <06> Undergraduate
- <07> Masters
- <08> Doctorate or above
- <99> No answer

D6. What is your Hukou?

- Rural
- Non-rural
- Other

B. NEWS PREFERENCES AND ATTITUDES

D7. Where do you normally get your domestic and international news?

- Domestic websites

- International websites
- Local TV news
- National news
- Local satellite stations
- International satellite stations
- Municipal newspapers
- Provincial newspapers
- International newspapers
- Magazines
- Social media
- Other

D8: How much attention do you pay to political news?

- <1> A lot of attention
- <2> Some attention
- <3> Not much attention
- <4> No attention at all
- <99> No answer

D9. Please choose which of the following news stories you would like to read:

1. "The US condemns China's COVID response"
2. "Guangzhou's foreign students receive the COVID vaccine."
3. "Monster movies are losing their appeal"

D10: In general, how do you feel towards the United States government?

- <1> Very favourable
- <2> Favourable
- <3> Unfavourable
- <4> Very unfavourable
- <99> No answer

D11: In general, how do you feel towards American people?

- <1> Very favourable
- <2> Favourable
- <3> Unfavourable
- <4> Very unfavourable
- <99> No answer

D12: In general, would you consider your political ideology to be very conservative, quite conservative, quite liberal, or very liberal?

- <1> Very conservative
- <2> Quite conservative
- <3> Quite liberal
- <4> Very liberal
- <99> No answer

D13. To what extent to you agree with the below sentence: "If I could choose any nationality, I would still choose to be Chinese"

- <1> Strongly agree
- <2> Agree to some extent
- <3> Disagree to some extent

- <4> Strongly disagree

D14. Please answer the following general knowledge question: Who is the United States' Senate Majority leader?

- Mitch McConnell
- Nancy Pelosi
- Ted Cruz
- Chuck Schumer

C. EXPERIMENT

Participants are randomly allocated to either story 1 or story 2. Depending on their choice in D9, they are given one of the following prompts (a) or (b)

(a) Please read the following:

OR

(b) In order to examine your response to different content, you have been randomly allocated a different news article to your choice. Please read the following:

STORY 1:

The US condemns China's COVID response

China Youth News

2021-05-15 1:25

According to multiple U.S. media reports, on May 14 the U.S. once again blamed China for the COVID epidemic.

At the press conference, the U.S. Secretary of State harshly criticised China. He claimed that China not only concealed and failed to report the epidemic at the beginning, but also did not take any necessary measures against the epidemic, which led to the global spread of the COVID-19 virus. He said: "China should be held responsible for the COVID-19 epidemic".

STORY 2:

Monster movies are losing their appeal

China Youth News

2021-05-15 1:25

More than ten days after its release, "Godzilla vs. King Kong" finally surpassed 1 billion yuan at the Chinese box office. Previous monster movies, "Godzilla" and "Godzilla 2: King of the Monsters" did not break 1 billion. The domestic box office of "King Kong: Skull Island" is 1.158 billion, the highest so far.

But compared to this year's Spring Festival domestic films that exceeded one billion in a single day, it looks like the genre of monster movies is not that attractive to the Chinese audience anymore.

D. OUTCOME QUESTIONS

D15: Please briefly write your thoughts on the news story:

PART III: Please answer the following to the best of your ability. Your answers will remain anonymous

D16: For each of the below, please tell me how much trust you have in them.

i. Chinese local Government

No trust										Full trust
0	10	20	30	40	50	60	70	80	90	100

- <99> No answer

ii. Chinese central Government

No trust										Full trust
0	10	20	30	40	50	60	70	80	90	100

- <99> No answer

D17: In your opinion, how well did the following deal with the outbreak of COVID in January 2020?

b) Wuhan local government
c) Chinese central government
d) US government

Extremely badly										Extremely well
0	10	20	30	40	50	60	70	80	90	100

- <99> No answer

D18: To what extent do you agree with the following:

a) In general, China is the best country in the world
b) China is a democratic country
c) The UN should launch an independent inquiry into the U.S.'s handling of the COVID outbreak
d) The UN should launch an independent inquiry into China's initial handling of the COVID outbreak
e) Chinese newspapers should launch an independent inquiry into China's initial handling of the COVID outbreak
f) A Chinese commission should launch an independent inquiry into China's initial handling of the COVID outbreak

- <1> Strongly agree
- <2> Agree to some extent
- <3> Disagree to some extent
- <4> Strongly disagree
- <99> No answer

II. SAMPLE & ATTRIBUTES

Table A1: Attributes of the sample vs. online population (excluding no answer)

	% of sample	% of population (inc. <18s, except for 'Age') (2020) [1]	% Weibo users (2020 [2] unless stated. Inc. <18s, except 'Age')
**Gender**			
Male	58	51	51
Female	42	49	49
**Age**			
18-24	12	7 (20-24)	30 (<24)
25-34	52	20	48 (24-33)
35-44	26	18	18 (34-43)
45-54	8	22	3 (44-53)
55+	3	33	1 (>54)
**Ethnicity**			
Han	97	98	*Not available*
Non-Han	3	2	
**Education**			
No higher education	19	84	24 *(in 2017[3])*
Higher education	81	16	76
**City-province**			
Yes	31	7	*Not available*
No	69	93	
**Urban hukou**			
Yes	86	64	*Not available*
No	14	36	

Table A2: Balance across experimental conditions. Difference reported is a two-way t-test. Standard deviations reported in brackets. ***p<0.01; **p<0.05; *p<0.1.

	**N**	**Control Mean [SE]**	**N**	**Treatment Mean [SE]**	**t-test (1)-(2)**
**Choice of criticism article**	580	0.348	583	0.334	0.014
		[0.020]		[0.020]	
**Sex**	580	1.607	583	1.557	0.049*
		[0.020]		[0.021]	
**Urban**	580	0.333	583	0.285	0.048*
		[0.020]		[0.019]	
**Age group**	580	3.417	583	3.346	0.071
		[0.037]		[0.037]	
**Han**	580	1.026	583	1.029	-0.003
		[0.007]		[0.007]	
**Education level**	579	5.846	583	5.851	-0.004
		[0.028]		[0.029]	
**Urban Hukou**	574	1.136	578	1.151	-0.015
		[0.014]		[0.015]	
**Social media user**	580	0.812	583	0.828	-0.016
		[0.016]		[0.016]	
**Frequency of news reading**	578	2.249	582	2.199	0.050
		[0.044]		[0.042]	
**Frequency of international news reading**	580	0.560	583	0.532	0.029
		[0.021]		[0.021]	
**Attitude to US government**	576	31.245	575	31.663	-0.418
		[1.057]		[1.042]	
**Attitude to US people**	576	45.248	574	45.232	0.017
		[1.084]		[1.099]	
**Left-right ideology**	575	3.337	580	3.364	-0.026
		[0.036]		[0.036]	
**Nationalism (reversed)**	573	1.319	579	1.421	-0.102***
		[0.024]		[0.029]	
**Knowledge of US politics**	580	0.188	583	0.180	0.008
		[0.016]		[0.016]	
**Region of China**	537	2.551	546	2.535	0.016

[1] http://www.stats.gov.cn/sj/ndsj/2021/indexeh.htm
[2] https://data.weibo.com/report/reportDetail?id=456
[3] Medaglia, R., & Zhu, D. (2017). Public deliberation on government-managed social media: A study on Weibo users in China. *Government Information Quarterly*, 34(3), 533-544

III. <u>READING CHOICE</u>

Table A1: Balance table on preference for reading about entertainment story. For ease of interpretation and comparison all non-dichotomous variables are broken down into dichotomous variables for those who responded above and below the mean value. Difference reported is a two-way t-test. Standard deviations reported in brackets. ***p<0.01; **p<0.05; *p<0.1.

	Chose entertainment		Did not choose entertainment		Difference Pairwise t-test	
Variable	N	Mean/(Var)	N	Mean/(Var)	N	P-value
High nationalism	1011	0.695	152	0.691	1163	0.909
		(0.212)		(0.215)		
Like US government	1011	0.424	152	0.553	1163	0.003***
		(0.245)		(0.249)		
Like US people	1011	0.523	152	0.572	1163	0.258
		(0.250)		(0.246)		
Liberal political ideology	1011	0.455	152	0.368	1163	0.045**
		(0.248)		(0.234)		
High news from social media	1011	0.823	152	0.803	1163	0.543
		(0.146)		(0.159)		
High news frequency	1011	0.323	152	0.428	1163	0.011**
		(0.219)		(0.246)		
High news interest	1011	0.553	152	0.500	1163	0.222
		(0.247)		(0.252)		
Good knowledge of US politics	1011	0.181	152	0.204	1163	0.497
		(0.148)		(0.163)		
Male	1011	0.583	152	0.579	1163	0.932
		(0.243)		(0.245)		
City province	1011	0.300	152	0.368	1163	0.087*
		(0.210)		(0.234)		
Over 35	1011	0.366	152	0.349	1163	0.680
		(0.232)		(0.229)		
Han ethnicity	1010	0.975	151	0.980	1161	0.715
		(0.024)		(0.020)		
Undergraduate degree	1011	0.807	152	0.803	1163	0.896
		(0.156)		(0.159)		
Urban Hukou	1003	0.850	149	0.899	1152	0.112
		(0.127)		(0.091)		

Table A2: Balance table on preference for reading about neutral news story. For ease of interpretation and comparison all non-dichotomous variables are broken down into dichotomous variables for those who responded above and below the mean value. Difference reported is a two-way t-test. Standard deviations reported in brackets. ***p<0.01; **p<0.05; *p<0.1.

	Chose Covid news		Did not choose Covid news		Difference Pairwise t-test	
Variable	N	Mean/(Var)	N	Mean/(Var)	N	P-value
High nationalism	549	0.636	614	0.748	1163	0.000***
		(0.232)		(0.189)		
Like US government	549	0.468	614	0.417	1163	0.079*
		(0.249)		(0.243)		
Like US people	549	0.559	614	0.503	1163	0.056*
		(0.247)		(0.250)		
Liberal political ideology	549	0.444	614	0.443	1163	0.960
		(0.247)		(0.247)		
High news from social media	549	0.834	614	0.808	1163	0.242
		(0.139)		(0.156)		
High news frequency	549	0.352	614	0.324	1163	0.323
		(0.228)		(0.219)		
High news interest	549	0.541	614	0.550	1163	0.745
		(0.249)		(0.248)		
Good knowledge of US politics	549	0.191	614	0.178	1163	0.547
		(0.155)		(0.146)		
Male	549	0.565	614	0.598	1163	0.254
		(0.246)		(0.241)		
City province	549	0.322	614	0.296	1163	0.339
		(0.219)		(0.209)		
Over 35	549	0.377	614	0.352	1163	0.372
		(0.235)		(0.228)		
Han ethnicity	548	0.978	613	0.974	1161	0.642
		(0.021)		(0.025)		
Undergraduate degree	549	0.816	614	0.798	1163	0.439
		(0.150)		(0.161)		
Urban Hukou	543	0.884	609	0.833	1152	0.013**
		(0.103)		(0.140)		

Figure A1: Marginal effects of independent variables on choice to read foreign shaming. All variables standardised from 0-1. Adjusted logistic regression with all variables included. Horizontal lines denote 90% confidence intervals.

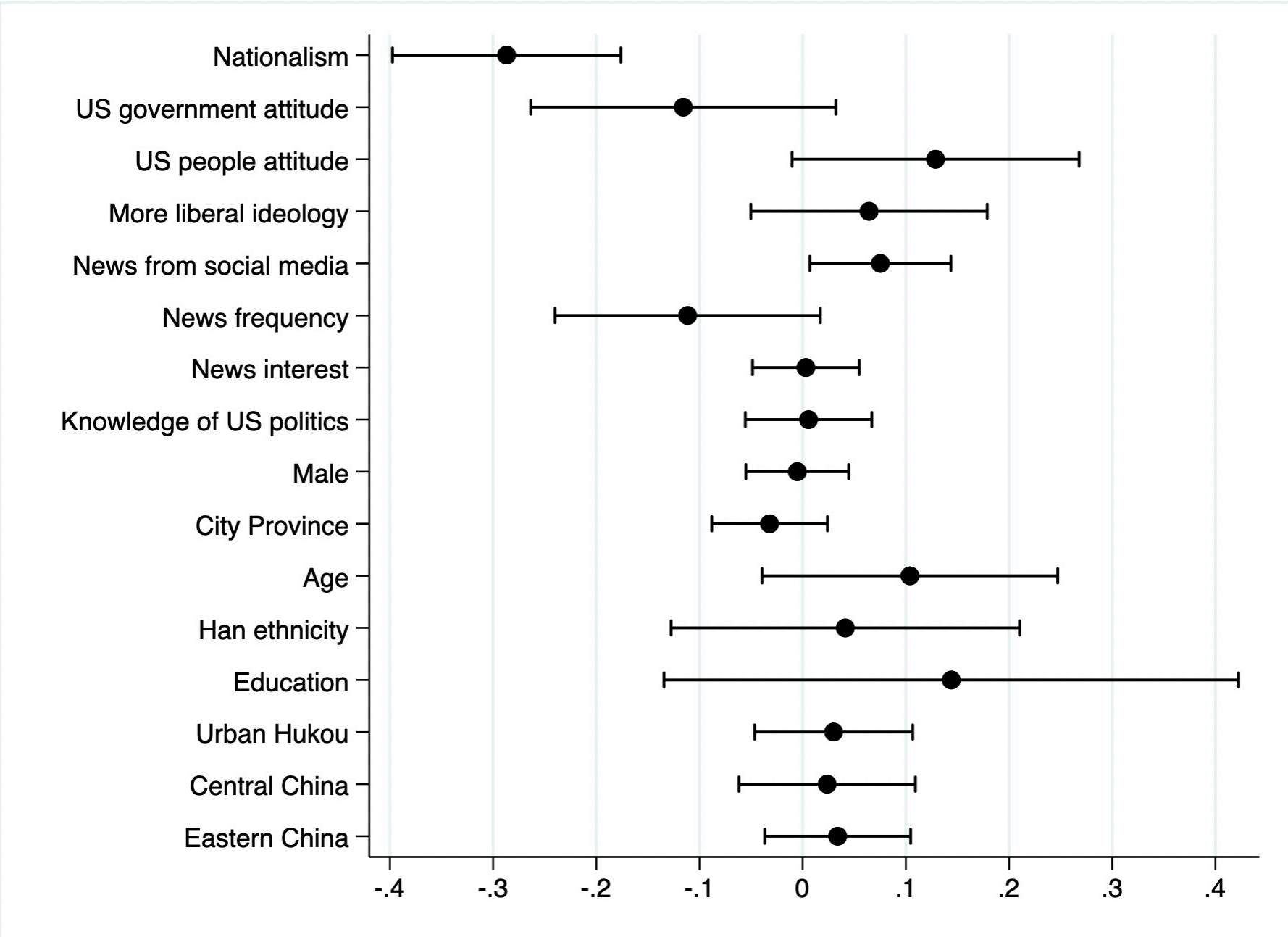

Figure A2: Marginal effects of independent variables on choice to read entertainment story. All variables standardised from 0-1, Adjusted logistic regression with all variables included. Horizontal lines denote 90% confidence intervals.

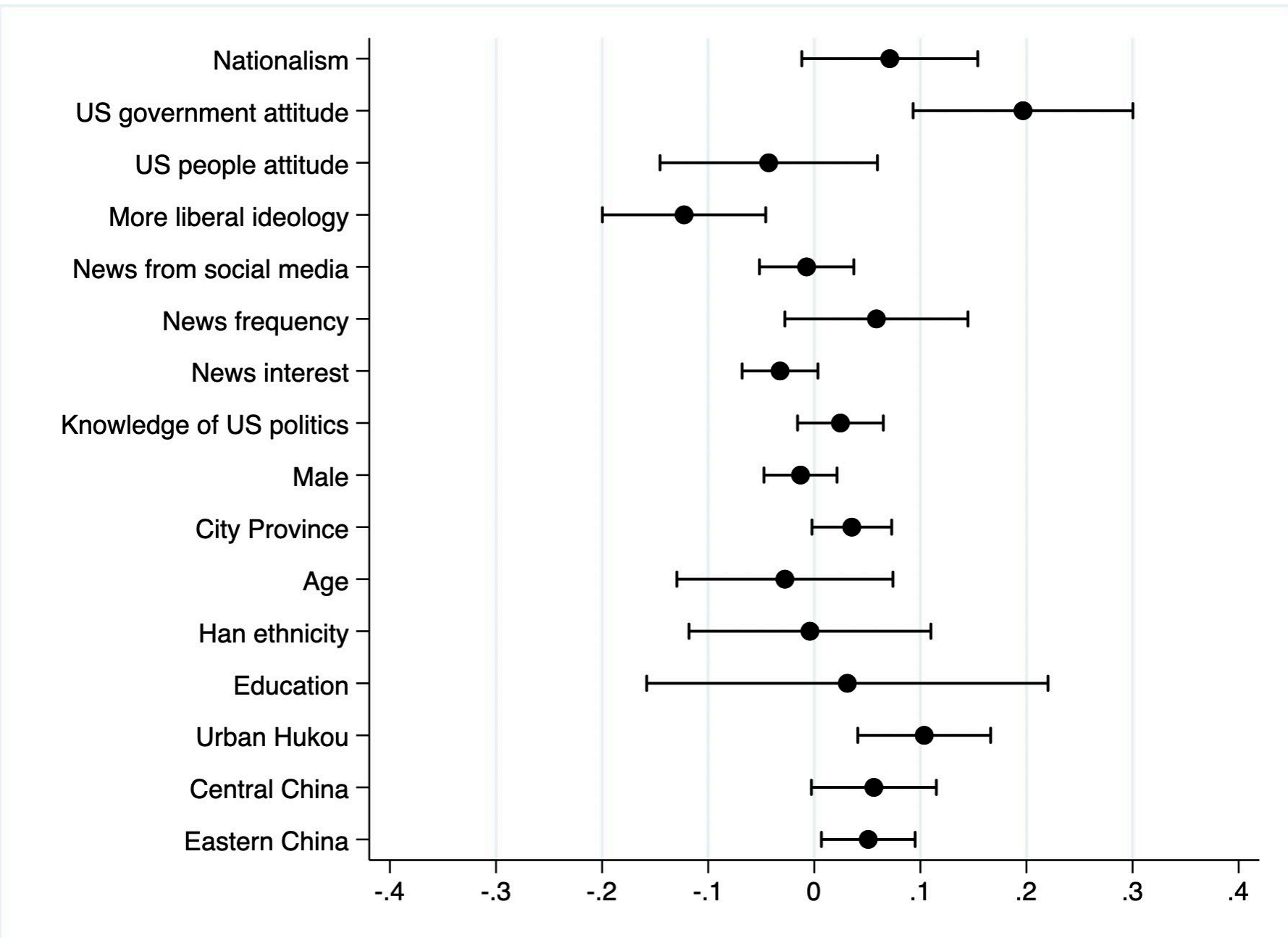

Figure A3: Marginal effects of independent variables on choice to read neutral news story. All variables standardised from 0-1, Adjusted logistic regression with all variables included. Horizontal lines denote 90% confidence intervals.

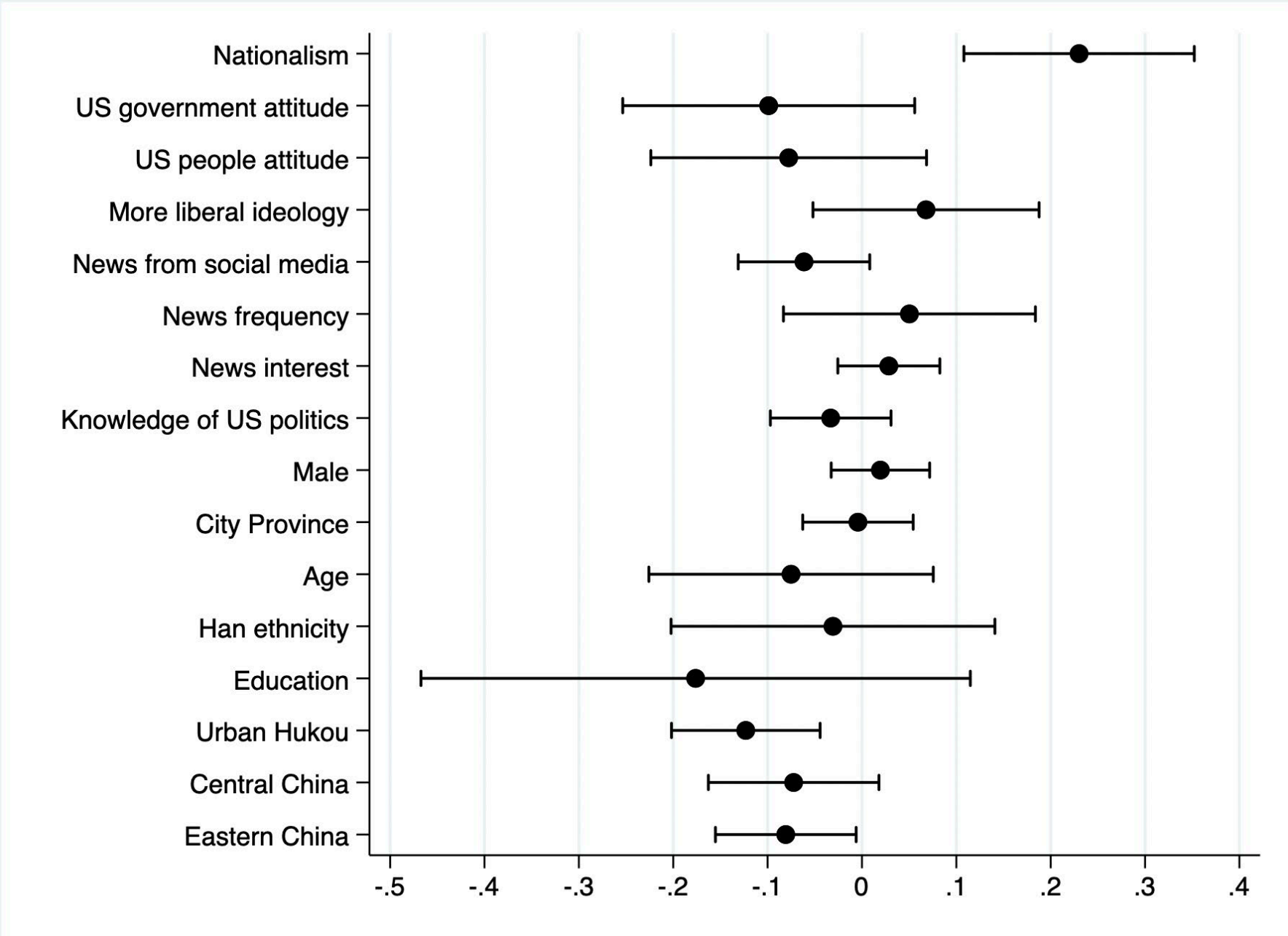

Note 1: Preferences to Read Foreign Shaming

As Figure A1 shows, the only variable to significantly influence people's choice to read about criticism is nationalism. The only other variable that even (weakly) significantly affects this choice is people's use of social media for news. This could indicate previous exposure to news about criticism (although there are no impacts from interest in news, frequency of exposure, or knowledge about the US), or more openness to other views (liberal ideology and liking American people were also non-significantly positive).

Perhaps the most interesting finding, however, is that except for nationalism, almost none of the variables showed any significant impact on the choice to read criticism. This is also the case in similar studies on Chinese people's choice to read foreign news (Huang & Yeh 2017) and is likely to be for two main reasons. Firstly, we deliberately isolated criticism as the main difference between the two news headlines. The headlines were on the same topic, and both included reference to foreign entities. We gave very little extra information, such that the main aspect that respondents were responding to was criticism. As noted, COVID-19 and American criticism of COVID-19 were prominent in Chinese news prior to this survey, and so demographic characteristics would not have affected previous knowledge of the issue and the criticism. The sheer importance of nationalism in determining preferences towards criticism means that this variable likely dominated other nuances in people's responses. Moreover, we did not ask pre-treatment for people to evaluate their regime (as we asked this post-treatment), which is one variable that has been shown to influence reading preferences for foreign news (Huang & Yeh 2017).

Secondly, the inclusion of an entertainment headline may have diluted the impact of other demographic variables. Some variables connected to a more international outlook may influence both preferences to read criticism and preferences to read entertainment. To examine this, I

exclude entertainment from the analysis and examine the demographic differences between the 'news readers': those who chose to read about the critical COVID-19 story versus the neutral COVID-19 story. This is not a pure comparison as it selectively excludes certain groups of respondents, but may be instructive on the differences between the two.

As Figure A1a shows, there are still no big demographic differences (in geography, ethnicity, age, sex, or education), but there are significant differences coming from attitudes to the United States. Readers of criticism (compared to just news) are more liberal and more likely to say they like the American people, suggesting a more pro-West attitude drives a desire to read criticism. But they are also *less* likely to say they like the American government. This may reflect people who like the US government not wanting to hear it saying critical things about their country, or those who don't like the US government wanting to satisfy or confirm their dislike by reading about it being critical.

Figure A1a: Marginal effects of independent variables on choice to read foreign shaming versus news story only (excluding those who chose entertainment). All variables standardised from 0-1. Adjusted logistic regression with all variables included. Horizontal lines denote 90% confidence intervals.

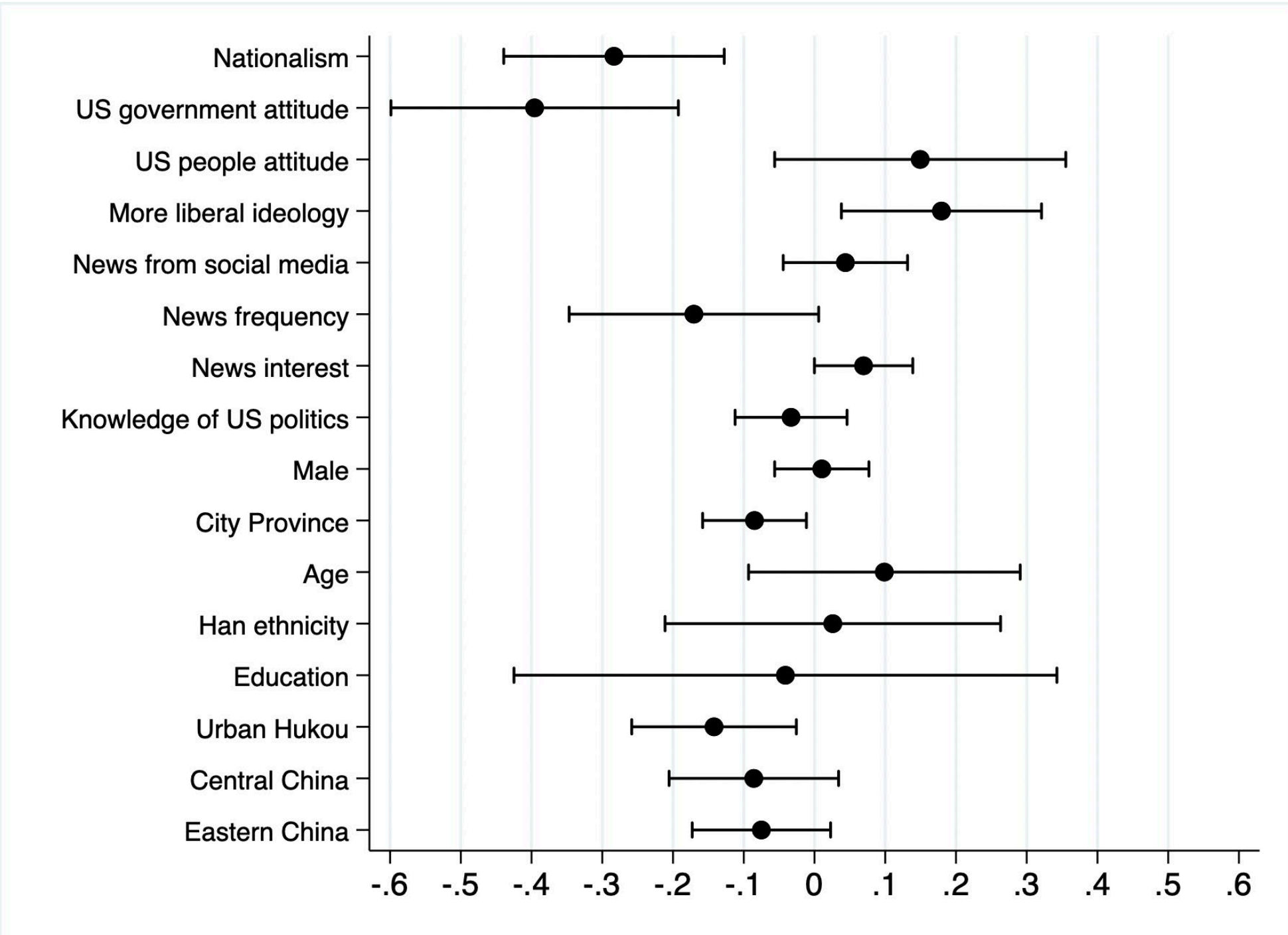

NOTE 2: Behavioural Choice Versus Stated Preference

Stated preferences are not the same as behavioural choices. In the study I asked respondents: "*Please choose which of the following news stories you would like to read*". This language was used to reflect previous studies that measure selective exposure. For example, Huang & Yeh's (2017: 622) article on Chinese people's choices to read foreign media gives respondents two stories and asks: "*If you were to read one of them, which would you choose*". Knox et al.'s (2019: 1540) article "Design, Identification, and Sensitivity Analysis for Patient Preference Trials", which is written to provide best practice for these kinds of experiments, asks for television news: "*Which of these programs would you like to watch now*". Following these articles, I made the assumption that respondents would take "*Please choose which of the following news stories you would like to read*" to mean that they were expecting to choose a story to read.

However, given that the survey asked respondents to choose a story they would "like to read" rather than to choose a story "to read", it is plausible that they considered that they were being asked to state a general preference rather than make a definitive choice. To test this, I carried out a small post-hoc study with 301 respondents in the United States in November 2025.

I firstly gave respondents the same prompt as the study: "**Please choose which one of the following recent news articles you would like to read**" with three headlines that closely resembled the study: one on Chinese criticism of human rights in the United States; one on general human rights in the United States; and one on movie trends in 2025. After some unrelated distractor questions, I then gave respondents the same headlines and a second prompt: **"Please choose one of the following recent news articles that you now want to read"**. This allows us to compare the original survey question to a question that was designed to as explicitly as possible ask respondents to make a choice. While this post-hoc survey was in the United States and was about human rights rather than about COVID-19, there is no reason to think that the dynamics of the two questions would not apply in the same way.

Of the 301 respondents, 281 gave an identical response to both cases, with only 20 giving a choice of story that was different to their original choice/preference. This gives a Cronbach's Alpha of 0.93 between the two questions (a value over 0.9 suggests that two items are testing the same construct in a different form (Tavakol & Dennick 2011)). This strongly supports our intuition that survey respondents did indeed judge that the study question was not just asking them to state their broader preference for news stories but was asking them to make a choice of which story they wanted to read.

IV. EFFECTS OF SHAMING

For all figures and tables: unless otherwise indicated: outcomes and covariates are standardised from 0-1, using adjusted OLS with covariates. Positive effect sizes denote more 'positive' response to questions and horizontal lines represent 90% confidence intervals for estimates.

Table A3: Average Conditional Treatment Effects of criticism by reading preference.: a) for those who chose to read an b) for those who chose not to. Outcomes are standardised from 0-1, using adjusted OLS with covariates, where higher value equates to more support for the variable. Horizontal lines represent 90% confidence intervals. Support for independent inquiry into the US's original COVID-19 response included for comparison purposes.

a) READERS

	Central gov COVID	Local gov COVID	Central gov general	Local gov general	China better	China democratic	News inquiry China	Civil inquiry China	UN inquiry China	UN inquiry USA
Criticism treatment	-0.00578	-0.00756	-0.00858	0.0175	0.00513	0.00508	0.00262	-0.00414	0.00475	-0.0253
	(0.0154)	(0.0230)	(0.0171)	(0.0208)	(0.0201)	(0.0257)	(0.0337)	(0.0340)	(0.0357)	(0.0226)
Male	0.0253	0.0199	0.0135	0.0276	0.0108	0.00468	0.0312	0.00801	0.0440	-0.0112
	(0.0168)	(0.0231)	(0.0180)	(0.0225)	(0.0209)	(0.0272)	(0.0345)	(0.0354)	(0.0371)	(0.0215)
City-province	-0.0165	-0.0405	-0.0415*	0.0125	0.00369	0.0348	0.0176	-0.00469	-0.00562	-0.0364
	(0.0204)	(0.0271)	(0.0231)	(0.0242)	(0.0247)	(0.0313)	(0.0417)	(0.0416)	(0.0421)	(0.0280)
Age	-0.0575	-0.0931	-0.0134	0.0201	-0.0573	-0.00813	-0.0638	-0.107	0.0511	0.0168
	(0.0495)	(0.0700)	(0.0548)	(0.0650)	(0.0824)	(0.0789)	(0.109)	(0.111)	(0.109)	(0.0644)
Han	0.0487	-0.0123	-0.0142	-0.111**	0.0491	0.0652	0.0389	-0.0711	-0.0410	0.155***
	(0.0447)	(0.0951)	(0.0374)	(0.0516)	(0.0710)	(0.0788)	(0.117)	(0.121)	(0.0916)	(0.0597)
Education	0.0118	0.128	-0.109	0.0672	-0.108	0.327*	-0.0190	0.194	0.0248	-0.168
	(0.0984)	(0.150)	(0.125)	(0.141)	(0.153)	(0.179)	(0.218)	(0.230)	(0.248)	(0.141)
Urban Hukou	-0.0196	-0.0172	-0.0231	-0.0309	-0.0585**	-0.0592	-0.0340	-0.0463	-0.0427	0.00766
	(0.0222)	(0.0360)	(0.0225)	(0.0312)	(0.0287)	(0.0391)	(0.0482)	(0.0472)	(0.0543)	(0.0332)
Social media use	0.0245	0.0152	0.0291	0.0335	0.0422	0.0327	-0.000751	-0.0242	-0.0700	0.0311
	(0.0260)	(0.0324)	(0.0280)	(0.0341)	(0.0323)	(0.0388)	(0.0481)	(0.0485)	(0.0542)	(0.0377)
News frequency	-0.0894	-0.0747	-0.125*	-0.149**	-0.0502	-0.0841	0.0147	-0.00271	0.0372	-0.0914
	(0.0578)	(0.0748)	(0.0676)	(0.0723)	(0.0565)	(0.0811)	(0.0952)	(0.102)	(0.103)	(0.0730)
International news	-0.00129	-0.0213	0.0106	-0.0122	-0.00386	0.0316	0.0138	-0.0361	0.00140	-0.0302
	(0.0165)	(0.0245)	(0.0196)	(0.0232)	(0.0222)	(0.0285)	(0.0344)	(0.0360)	(0.0384)	(0.0240)
USgov attitude	-0.000342	0.169*	-0.0367	0.0604	-0.0230	0.131	0.164	0.172	0.0102	-0.210***
	(0.0653)	(0.0911)	(0.0543)	(0.0715)	(0.0579)	(0.0843)	(0.112)	(0.120)	(0.131)	(0.0799)
US people attitude	-0.0142	-0.0848	0.0604	0.0604	-0.0714	-0.108	0.0473	0.112	0.203	0.0145
	(0.0547)	(0.0826)	(0.0505)	(0.0650)	(0.0622)	(0.0787)	(0.106)	(0.116)	(0.124)	(0.0722)
Ideology	0.0273	0.0371	0.0384	0.0125	0.0870	-0.0174	-0.0312	-0.00219	0.0319	0.0739
	(0.0354)	(0.0504)	(0.0419)	(0.0529)	(0.0551)	(0.0610)	(0.0833)	(0.0801)	(0.0883)	(0.0515)
Nationalism	0.389***	0.515***	0.383***	0.437***	0.523***	0.576***	-0.0205	0.0198	-0.102	0.0847*
	(0.0563)	(0.0573)	(0.0582)	(0.0560)	(0.0552)	(0.0647)	(0.0735)	(0.0753)	(0.0710)	(0.0496)
US knowledge	0.00131	0.00989	0.0295*	0.00391	0.0272	-0.0348	0.0279	-0.00634	-0.0117	0.00791
	(0.0195)	(0.0323)	(0.0171)	(0.0231)	(0.0237)	(0.0318)	(0.0434)	(0.0435)	(0.0480)	(0.0252)
East China	-0.00827	0.0540	0.0154	0.0467	0.0342	0.0649	-0.0320	-0.0143	-0.0699	-0.0339
	(0.0227)	(0.0440)	(0.0300)	(0.0401)	(0.0320)	(0.0482)	(0.0574)	(0.0622)	(0.0617)	(0.0389)
Central China	-0.0284	0.0287	0.0187	0.0413	0.0167	0.0261	-0.0239	-0.0202	-0.0460	0.0211
	(0.0189)	(0.0363)	(0.0270)	(0.0327)	(0.0314)	(0.0398)	(0.0410)	(0.0468)	(0.0517)	(0.0316)
Constant	0.548***	0.228	0.588***	0.339***	0.429***	-0.0396	0.479**	0.471**	0.540**	0.768***
	(0.0954)	(0.158)	(0.108)	(0.121)	(0.139)	(0.164)	(0.208)	(0.210)	(0.220)	(0.133)
Observations	354	355	355	355	354	353	354	354	354	354
R-squared	0.353	0.272	0.313	0.260	0.361	0.276	0.040	0.055	0.050	0.135

b) NON- READERS

	Central gov COVID	Local gov COVID	Central gov general	Local gov general	China better	China democratic	News inquiry China	Civil inquiry China	UN inquiry China	UN inquiry USA
Criticism treatment	0.0106	0.0167	0.0301***	0.0479***	0.0318**	0.0518***	0.0583**	0.0474**	0.0649***	0.0313**
	(0.00910)	(0.0161)	(0.0102)	(0.0135)	(0.0128)	(0.0163)	(0.0230)	(0.0235)	(0.0236)	(0.0154)
Male	0.00434	0.00500	0.00294	0.00848	0.00513	-0.0111	0.0275	0.0182	0.0292	-0.0340**
	(0.00946)	(0.0162)	(0.0105)	(0.0137)	(0.0136)	(0.0167)	(0.0243)	(0.0247)	(0.0247)	(0.0158)
City-province	-0.00609	-0.0248	-0.0219*	0.00648	0.00523	-0.00472	-0.0276	-0.0372	-0.0377	0.0198
	(0.0113)	(0.0191)	(0.0128)	(0.0152)	(0.0140)	(0.0188)	(0.0275)	(0.0279)	(0.0275)	(0.0175)
Age	0.0131	-0.0826	0.0618**	0.0239	0.00168	-0.183***	-0.0457	-0.0452	0.00230	0.108**
	(0.0264)	(0.0526)	(0.0289)	(0.0397)	(0.0428)	(0.0472)	(0.0723)	(0.0730)	(0.0705)	(0.0517)
Han	-0.0154	0.0631	0.0137	0.0399	0.125**	0.148**	-0.0239	-0.0690	-0.0574	-0.0281
	(0.0209)	(0.0462)	(0.0322)	(0.0500)	(0.0614)	(0.0740)	(0.0685)	(0.0738)	(0.0688)	(0.0450)
Education	0.0203	0.0551	-0.0341	0.0249	0.125	0.119	-0.0354	0.0462	0.0291	0.0695
	(0.0437)	(0.0889)	(0.0569)	(0.0818)	(0.0800)	(0.0911)	(0.125)	(0.122)	(0.131)	(0.0946)
Urban Hukou	-0.0204	-0.0193	-0.0290**	-0.0227	-0.0109	-0.0579**	-0.0742**	-0.0846**	-0.0805**	0.00801
	(0.0138)	(0.0235)	(0.0120)	(0.0186)	(0.0201)	(0.0248)	(0.0327)	(0.0339)	(0.0359)	(0.0238)
Social media use	-0.0211*	-0.0338	-0.0126	-0.0234	-0.00558	-0.0401*	0.0176	0.0213	0.0534*	0.0102
	(0.0116)	(0.0228)	(0.0133)	(0.0184)	(0.0172)	(0.0218)	(0.0319)	(0.0334)	(0.0316)	(0.0234)
News frequency	-0.0360	-0.0406	-0.0761***	-0.0818**	-0.0231	-0.0753*	0.0450	0.0420	-0.0104	-0.158***
	(0.0221)	(0.0415)	(0.0262)	(0.0355)	(0.0342)	(0.0411)	(0.0609)	(0.0612)	(0.0581)	(0.0458)
International news	-0.0137	0.0115	-0.00255	0.0122	-0.0240*	0.0179	0.0125	-0.000272	-0.00872	0.00138
	(0.00940)	(0.0167)	(0.0107)	(0.0143)	(0.0139)	(0.0170)	(0.0248)	(0.0257)	(0.0255)	(0.0174)
USgov attitude	-0.121***	0.0268	-0.113***	-0.0209	-0.0304	-0.0206	0.399***	0.393***	0.292***	-0.203***
	(0.0280)	(0.0511)	(0.0260)	(0.0400)	(0.0349)	(0.0460)	(0.0698)	(0.0675)	(0.0773)	(0.0612)
US people attitude	0.0917***	0.0158	0.0981***	0.0824**	-0.0123	-0.0240	-0.177***	-0.229***	-0.0303	0.0766
	(0.0245)	(0.0475)	(0.0252)	(0.0402)	(0.0336)	(0.0446)	(0.0674)	(0.0648)	(0.0718)	(0.0534)
Ideology	0.0358	0.0310	0.0395	0.0305	0.0487	0.0472	0.0523	0.102*	0.116*	0.104**
	(0.0257)	(0.0420)	(0.0248)	(0.0335)	(0.0323)	(0.0406)	(0.0589)	(0.0601)	(0.0614)	(0.0419)
Nationalism	0.247***	0.306***	0.296***	0.396***	0.437***	0.401***	-0.0626	-0.0933	-0.0890	0.148***
	(0.0368)	(0.0523)	(0.0387)	(0.0486)	(0.0503)	(0.0578)	(0.0647)	(0.0644)	(0.0585)	(0.0430)
US knowledge	-0.00343	0.00780	-0.0104	-0.0227	0.000264	-0.0217	-0.0218	-0.0232	-0.0250	0.00689
	(0.0126)	(0.0198)	(0.0136)	(0.0181)	(0.0140)	(0.0211)	(0.0296)	(0.0309)	(0.0298)	(0.0194)
East China	0.0176	-0.00643	0.0406**	0.00633	0.0500*	0.00143	0.0449	0.0502	0.0288	0.000992
	(0.0171)	(0.0320)	(0.0194)	(0.0257)	(0.0270)	(0.0294)	(0.0389)	(0.0416)	(0.0419)	(0.0273)
Central China	0.0236	0.0135	0.0427**	0.00780	0.0462*	-0.0354	0.0154	0.0408	-0.00386	-0.00327
	(0.0160)	(0.0285)	(0.0179)	(0.0218)	(0.0241)	(0.0255)	(0.0333)	(0.0359)	(0.0339)	(0.0232)
Constant	0.696***	0.439***	0.601***	0.328***	0.253**	0.356***	0.501***	0.533***	0.374***	0.625***
	(0.0550)	(0.0975)	(0.0623)	(0.0893)	(0.112)	(0.114)	(0.128)	(0.141)	(0.129)	(0.0952)
Observations	685	685	685	683	682	674	682	682	682	681
R-squared	0.203	0.110	0.243	0.209	0.275	0.207	0.102	0.093	0.101	0.142

Table A4: Average Conditional Treatment Effects of criticism by reading preference, questions combined along compliance-backlash pathways. Support for inquiry into the US response not included. Outcomes standardised between 0-1, adjusted OLS with covariates, where higher value equates to more support for the variable. 90% confidence intervals.

	**(A) READERS**			**(B) NON-READERS**		
	(1)	(2)	(3)	(1)	(2)	(3)
	COVID response	Country/ government	China inquiry	COVID response	Country/ government	China inquiry
Criticism treatment	-0.00706	0.00653	0.00108	0.0136	0.0421***	0.0569***
	(0.0171)	(0.0155)	(0.0300)	(0.0112)	(0.00897)	(0.0203)
Male	0.0222	0.0152	0.0278	0.00467	0.000717	0.0250
	(0.0178)	(0.0162)	(0.0311)	(0.0114)	(0.00914)	(0.0215)
City-province	-0.0281	0.00253	0.00244	-0.0154	-0.00552	-0.0342
	(0.0211)	(0.0188)	(0.0365)	(0.0136)	(0.0102)	(0.0240)
Age	-0.0758	-0.0124	-0.0400	-0.0348	-0.0212	-0.0296
	(0.0530)	(0.0495)	(0.0960)	(0.0351)	(0.0271)	(0.0633)
Han	0.0175	-0.00303	-0.0244	0.0238	0.0788**	-0.0501
	(0.0646)	(0.0508)	(0.0967)	(0.0277)	(0.0377)	(0.0615)
Education	0.0733	0.0333	0.0667	0.0377	0.0628	0.0133
	(0.109)	(0.114)	(0.203)	(0.0584)	(0.0542)	(0.110)
Urban Hukou	-0.0186	-0.0434*	-0.0410	-0.0199	-0.0311**	-0.0798***
	(0.0274)	(0.0221)	(0.0417)	(0.0165)	(0.0121)	(0.0294)
Social use media	0.0197	0.0342	-0.0317	-0.0275*	-0.0206	0.0308
	(0.0257)	(0.0245)	(0.0439)	(0.0153)	(0.0127)	(0.0284)
News frequency	-0.0811	-0.0973*	0.0164	-0.0383	-0.0614**	0.0255
	(0.0609)	(0.0543)	(0.0879)	(0.0286)	(0.0253)	(0.0520)
International news	-0.0117	0.00773	-0.00696	-0.00109	0.00221	0.00118
	(0.0182)	(0.0168)	(0.0307)	(0.0116)	(0.00926)	(0.0221)
US government attitude	0.0843	0.0417	0.116	-0.0473	-0.0457*	0.361***
	(0.0658)	(0.0513)	(0.107)	(0.0347)	(0.0250)	(0.0602)
US people attitude	-0.0492	-0.0205	0.121	0.0538*	0.0334	-0.145**
	(0.0584)	(0.0462)	(0.102)	(0.0312)	(0.0236)	(0.0579)
Ideology	0.0326	0.0340	-0.000505	0.0334	0.0470**	0.0903*
	(0.0377)	(0.0405)	(0.0718)	(0.0307)	(0.0235)	(0.0517)
Nationalism	0.451***	0.484***	-0.0343	0.276***	0.399***	-0.0816
	(0.0506)	(0.0474)	(0.0625)	(0.0415)	(0.0366)	(0.0545)
US knowledge	0.00588	0.00689	0.00328	0.00218	-0.0126	-0.0233
	(0.0232)	(0.0168)	(0.0388)	(0.0143)	(0.0114)	(0.0271)
East China	0.0228	0.0448	-0.0388	0.00557	0.0250	0.0413
	(0.0296)	(0.0272)	(0.0519)	(0.0219)	(0.0176)	(0.0350)
Central China	-0.000404	0.0303	-0.0300	0.0186	0.0143	0.0175
	(0.0245)	(0.0226)	(0.0391)	(0.0204)	(0.0161)	(0.0291)
Constant	0.387***	0.323***	0.497***	0.567***	0.366***	0.469***
	(0.113)	(0.104)	(0.187)	(0.0676)	(0.0696)	(0.117)
Observations	354	353	354	685	672	682
R-squared	0.354	0.423	0.053	0.161	0.383	0.121

Table A5: Average Conditional Treatment Effects of criticism for all respondents, questions combined along compliance-backlash pathways. Support for inquiry into the US response not included. Outcomes standardised between 0-1, adjusted OLS with covariates, where higher value equates to more support for the variable. 90% confidence intervals.

	(1)<br>Covid response	(2)<br>Country/<br>government	(3)<br>China inquiry
Criticism treatment	0.00696	0.0297***	0.0387**
	(0.00931)	(0.00795)	(0.0167)
Chose to read criticism	-0.0180*	-0.0303***	0.0372**
	(0.00993)	(0.00908)	(0.0181)
Male	0.00974	0.00444	0.0276
	(0.00973)	(0.00829)	(0.0174)
City-province	-0.0192*	-0.00237	-0.0271
	(0.0115)	(0.00919)	(0.0195)
Age	-0.0465	-0.0166	-0.0371
	(0.0296)	(0.0245)	(0.0523)
Han	0.0234	0.0531*	-0.0421
	(0.0274)	(0.0312)	(0.0524)
Education	0.0314	0.0367	0.0189
	(0.0534)	(0.0513)	(0.0961)
Urban Hukou	-0.0150	-0.0327***	-0.0666***
	(0.0141)	(0.0109)	(0.0238)
Social use media	-0.0147	-0.00540	0.0104
	(0.0135)	(0.0120)	(0.0237)
News frequency	-0.0516*	-0.0704***	0.0251
	(0.0274)	(0.0246)	(0.0443)
International news	-0.00335	0.00523	0.000326
	(0.00974)	(0.00819)	(0.0180)
US government attitude	-0.0137	-0.0206	0.293***
	(0.0312)	(0.0232)	(0.0533)
US people attitude	0.0268	0.0177	-0.0666
	(0.0277)	(0.0213)	(0.0509)
Ideology	0.0239	0.0408*	0.0570
	(0.0240)	(0.0211)	(0.0416)
Nationalism	0.346***	0.433***	-0.0486
	(0.0332)	(0.0301)	(0.0405)
US knowledge	0.00214	-0.00608	-0.0134
	(0.0123)	(0.00962)	(0.0220)
East China	0.00712	0.0271*	0.0153
	(0.0176)	(0.0151)	(0.0290)
Central China	0.00822	0.0157	0.00406
	(0.0156)	(0.0131)	(0.0234)
Constant	0.519***	0.370***	0.459***
	(0.0595)	(0.0585)	(0.0989)
Observations	1,039	1,025	1,036
R-squared	0.231	0.402	0.087

Figure A4: Full Average Treatment Effects (regardless of reading preferences). Support for investigation into the US response is included as a comparison.

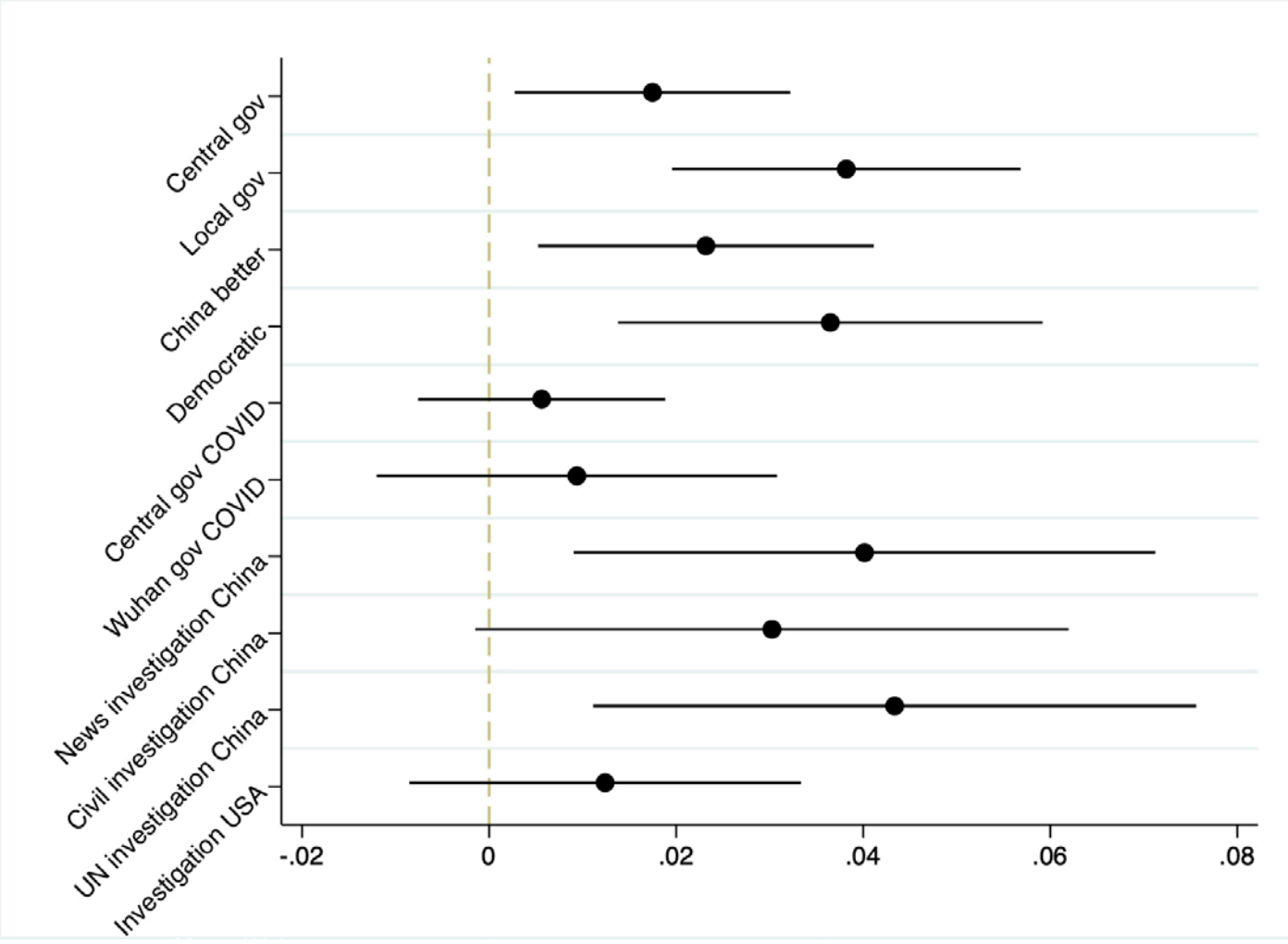

Figure A5: Difference between those who chose to read versus those who chose not to read (figure 2 in text). Support for investigation into the US response is included as a comparison

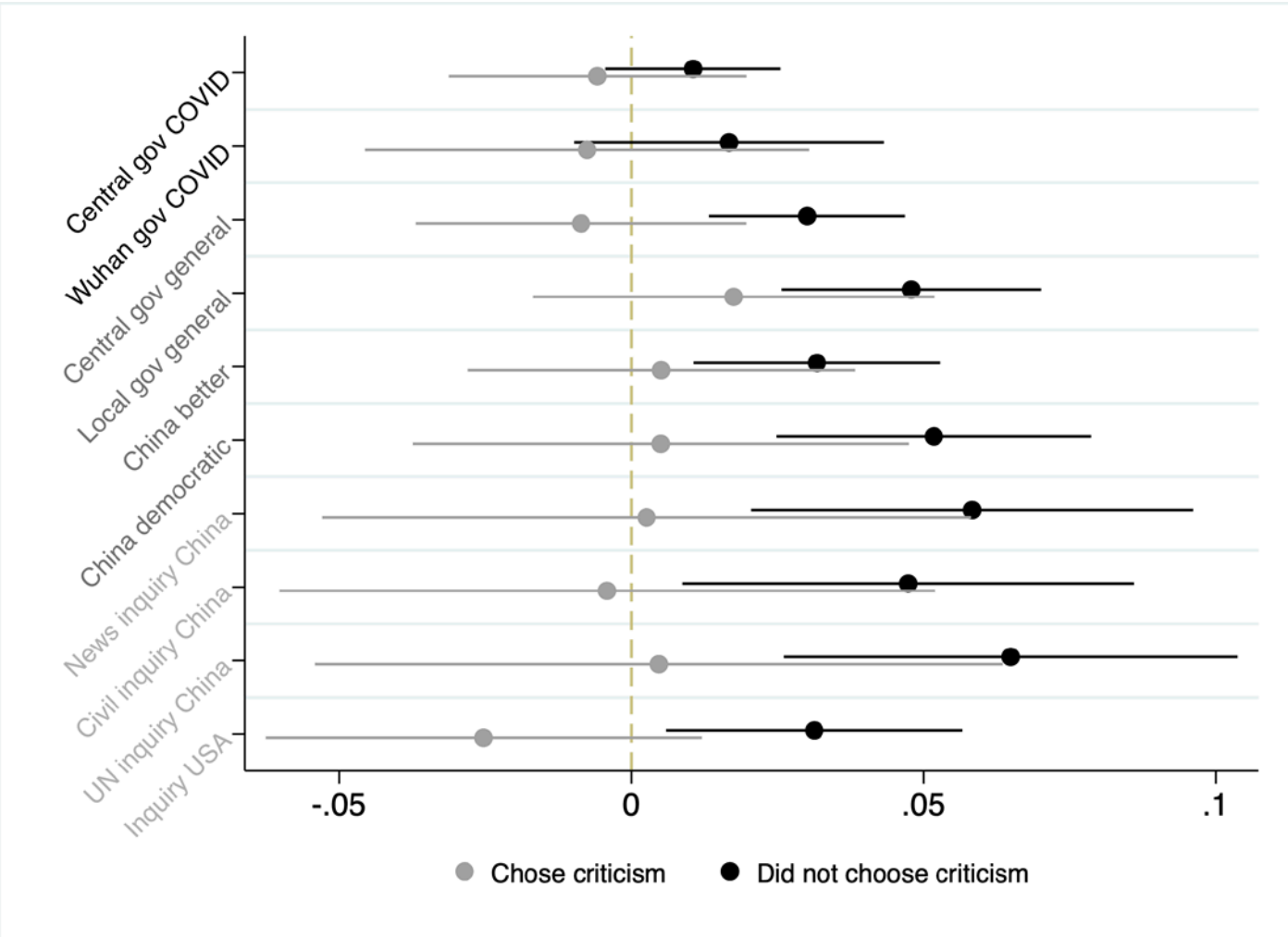

Figure A6: Not including nationalism as a control

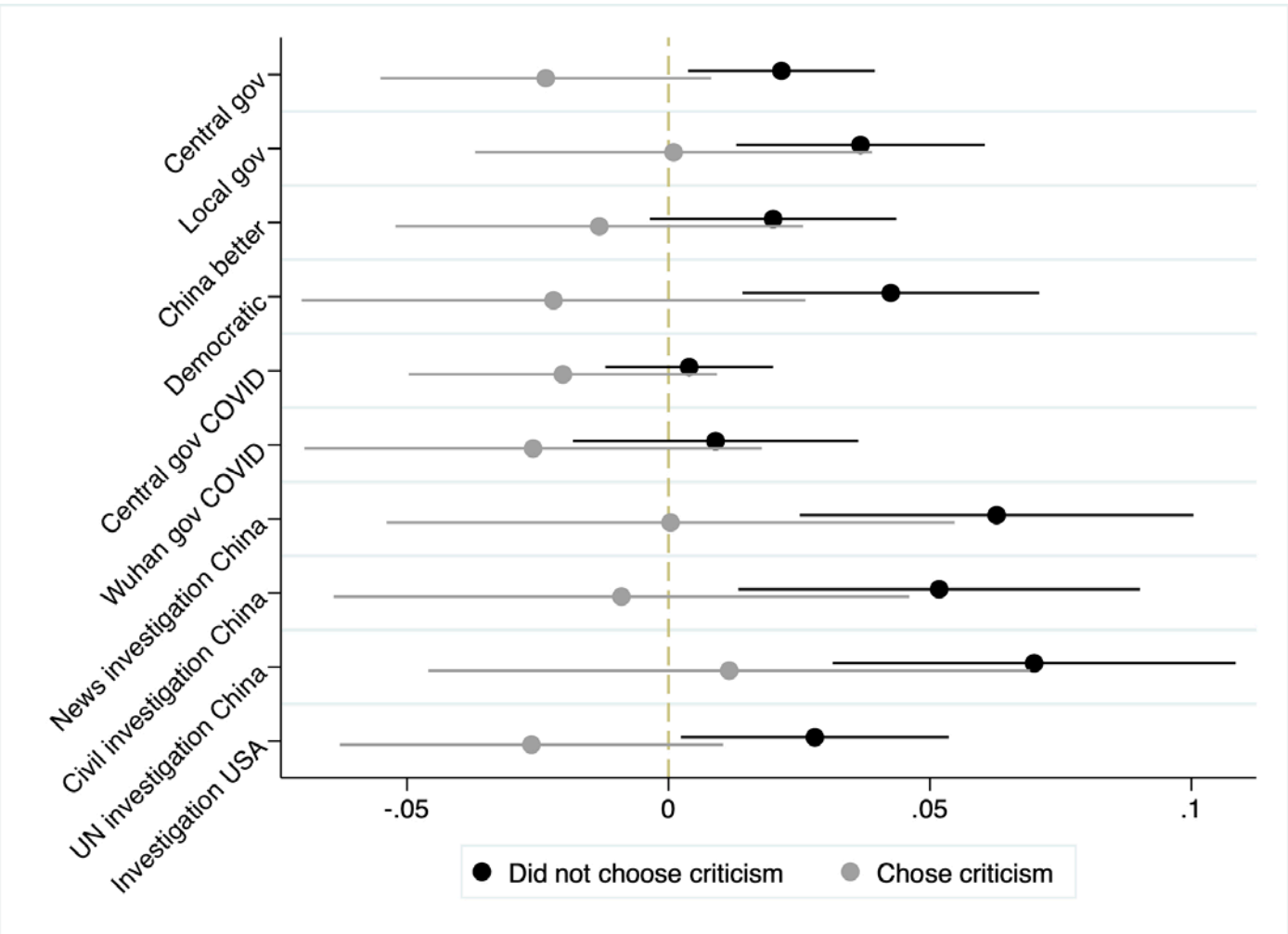

Figure A7: Unadjusted OLS without covariates, for all outcome variables

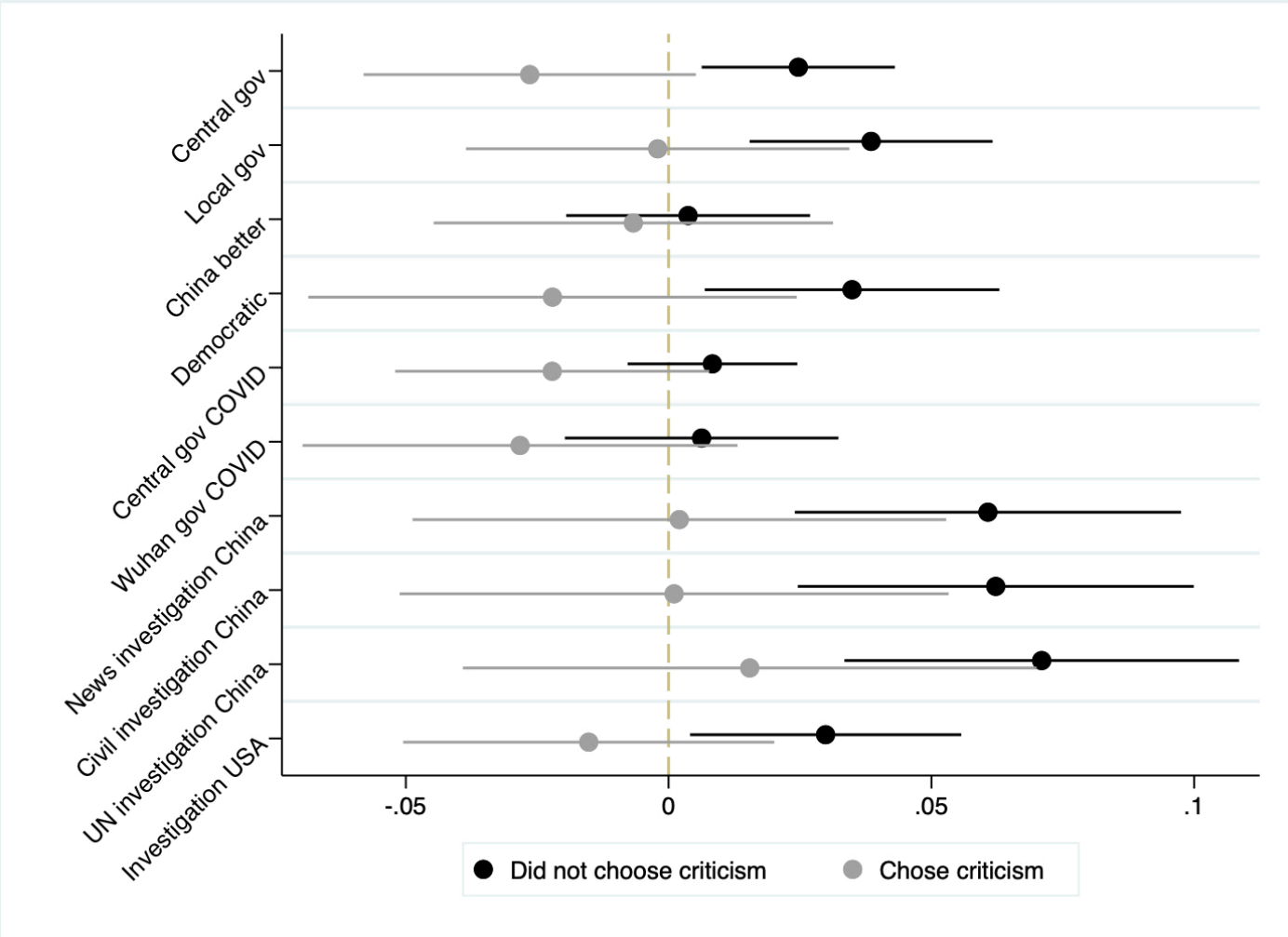

Figure A8: Results combined by pathway: (COVID response: Central gov COVID; Wuhan gov COVID. Country/government: Central; local; China better; China democratic. China investigations: all investigations in China). Figure 4 in text.

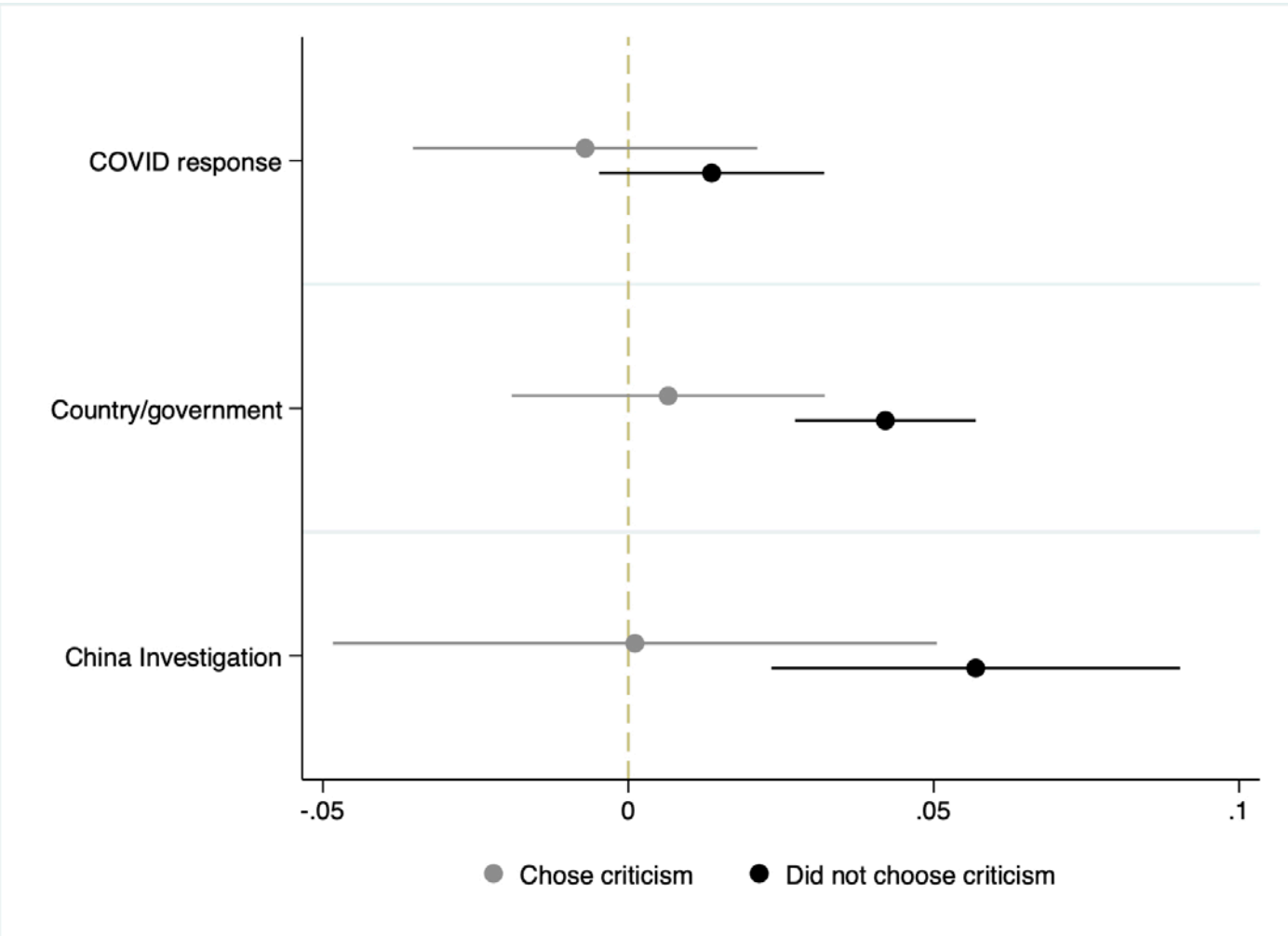

Figure A9: Not including nationalism as a control, for outcomes combined by pathway.

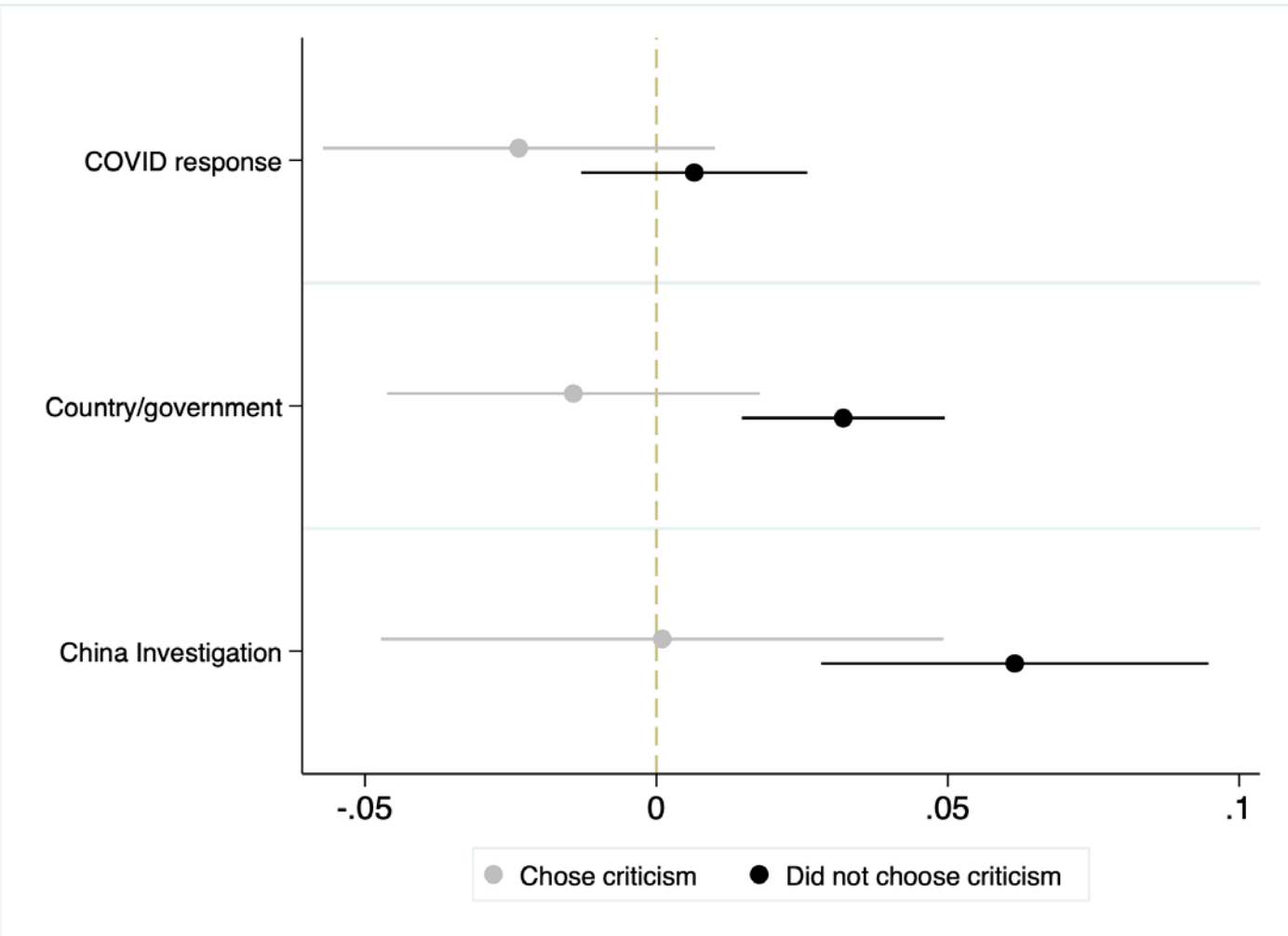

Figure A10: Unadjusted OLS without covariates, for outcomes combined by pathway.

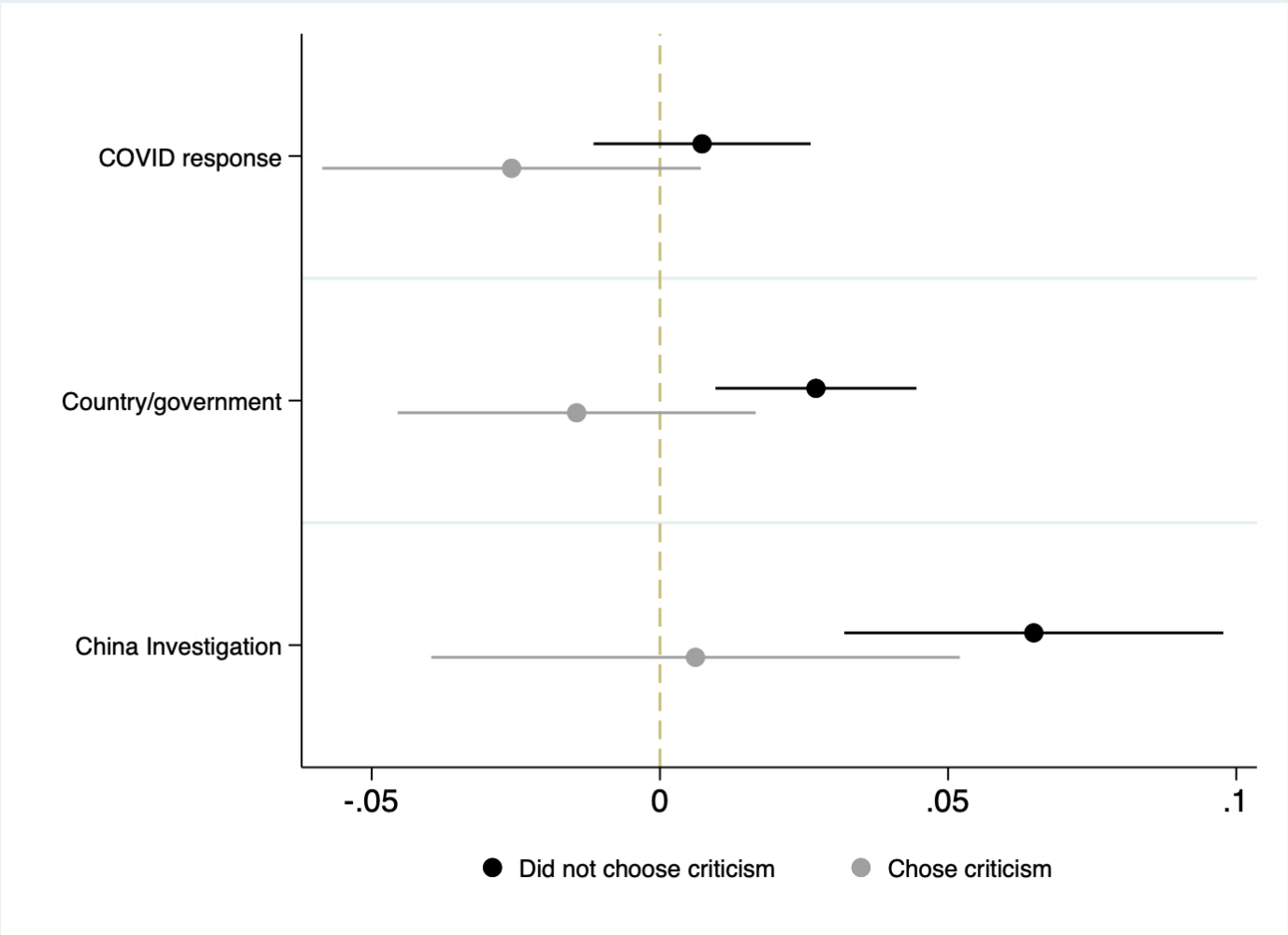

Figure A11: Excluding respondents who completed the whole survey in bottom-tenth percentile of time

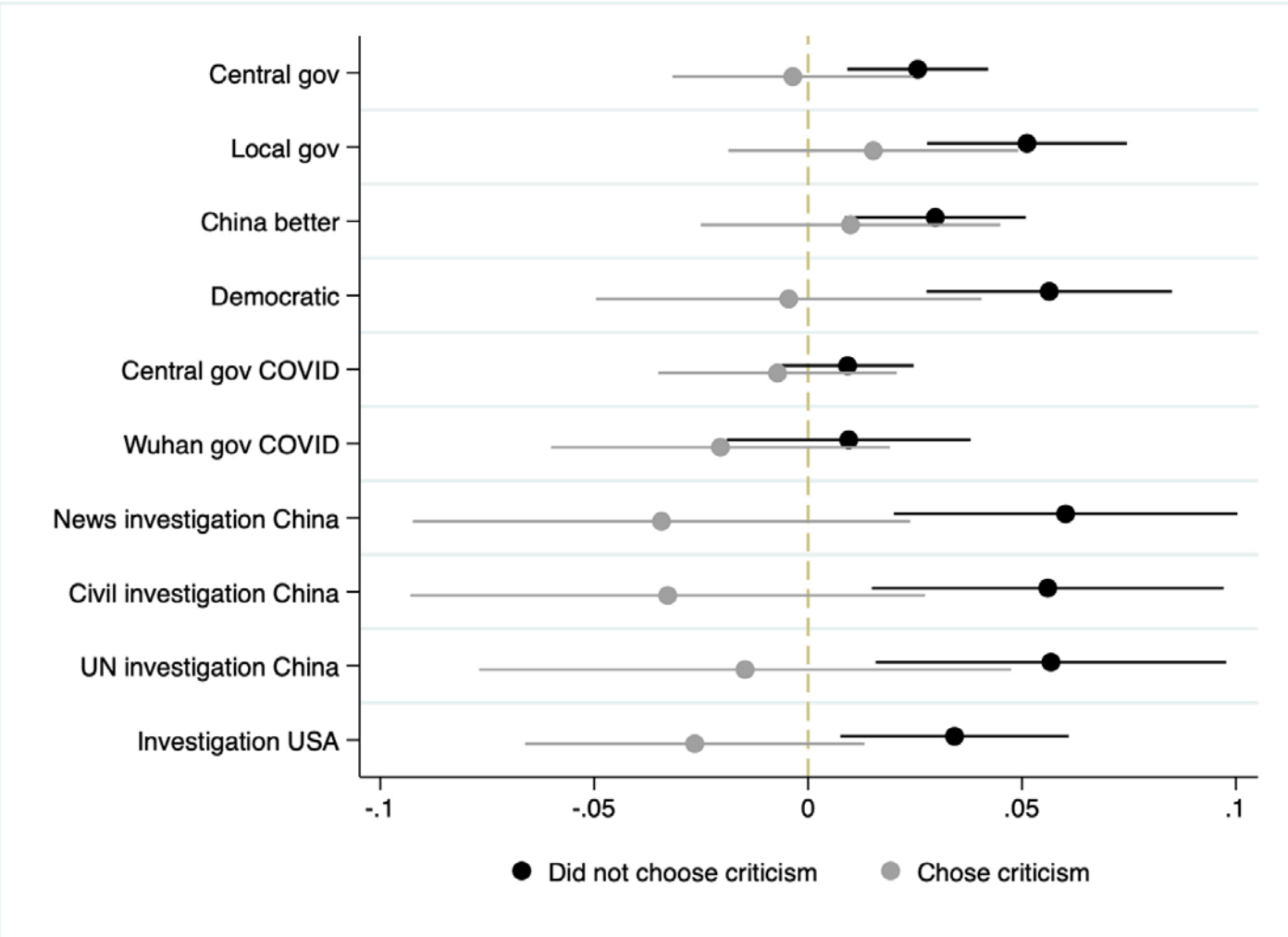

Figure A12: Results limited only to those who chose to read neutral news stories

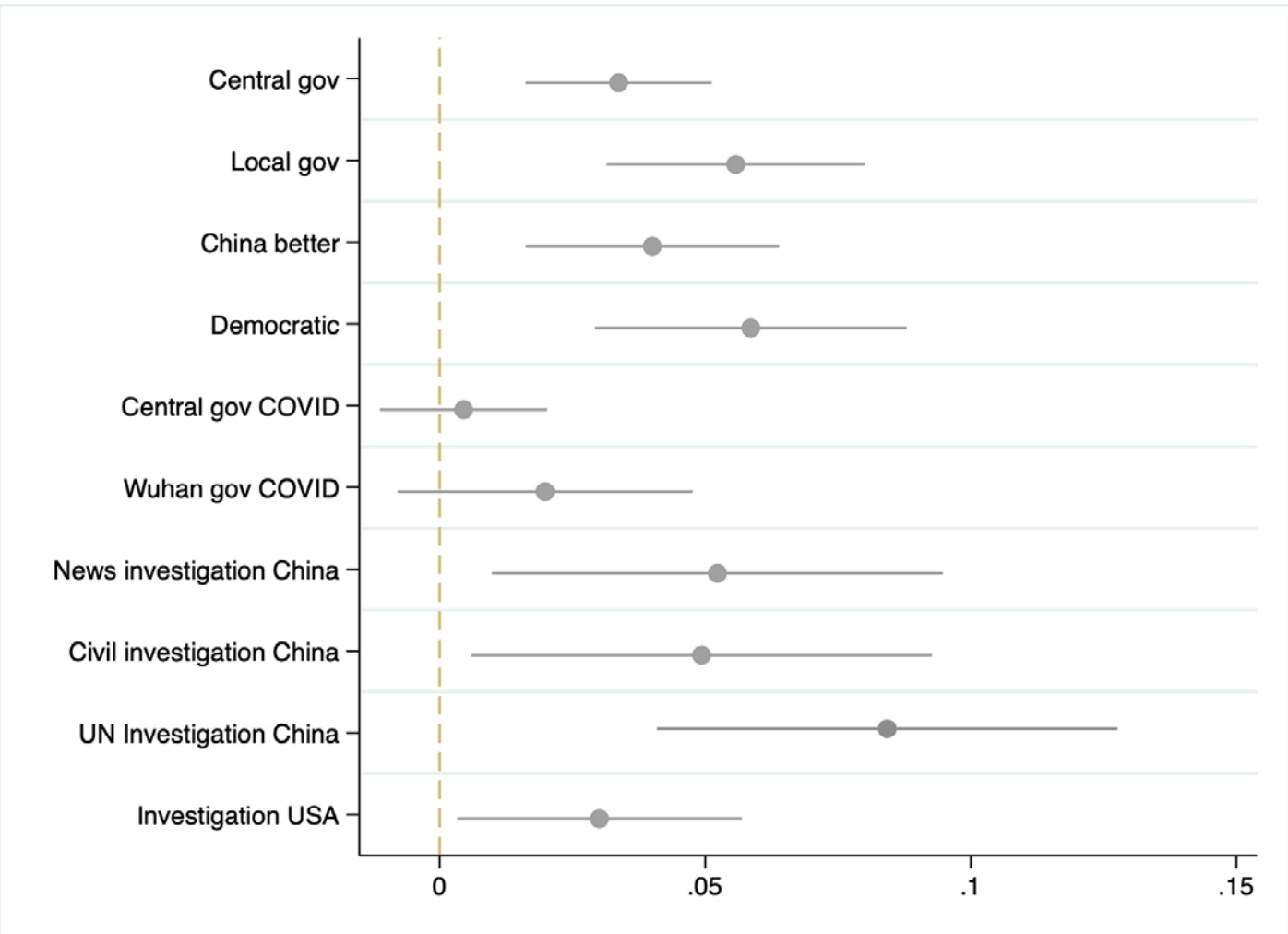

Figure A13: Comparison between full Average Treatment Effect and Treatment Effect **only** for those given their chosen story (comparing those who chose criticism and were given criticism, with those who did not choose criticism and were not given criticism. This approximates a 'choice' design).

These findings indicate that if people have freedom to select their news story, then those who read about criticism will end up holding similar attitudes to those who do not, except that they will be more willing to support an investigation (reflecting the differences in baseline support for an investigation).

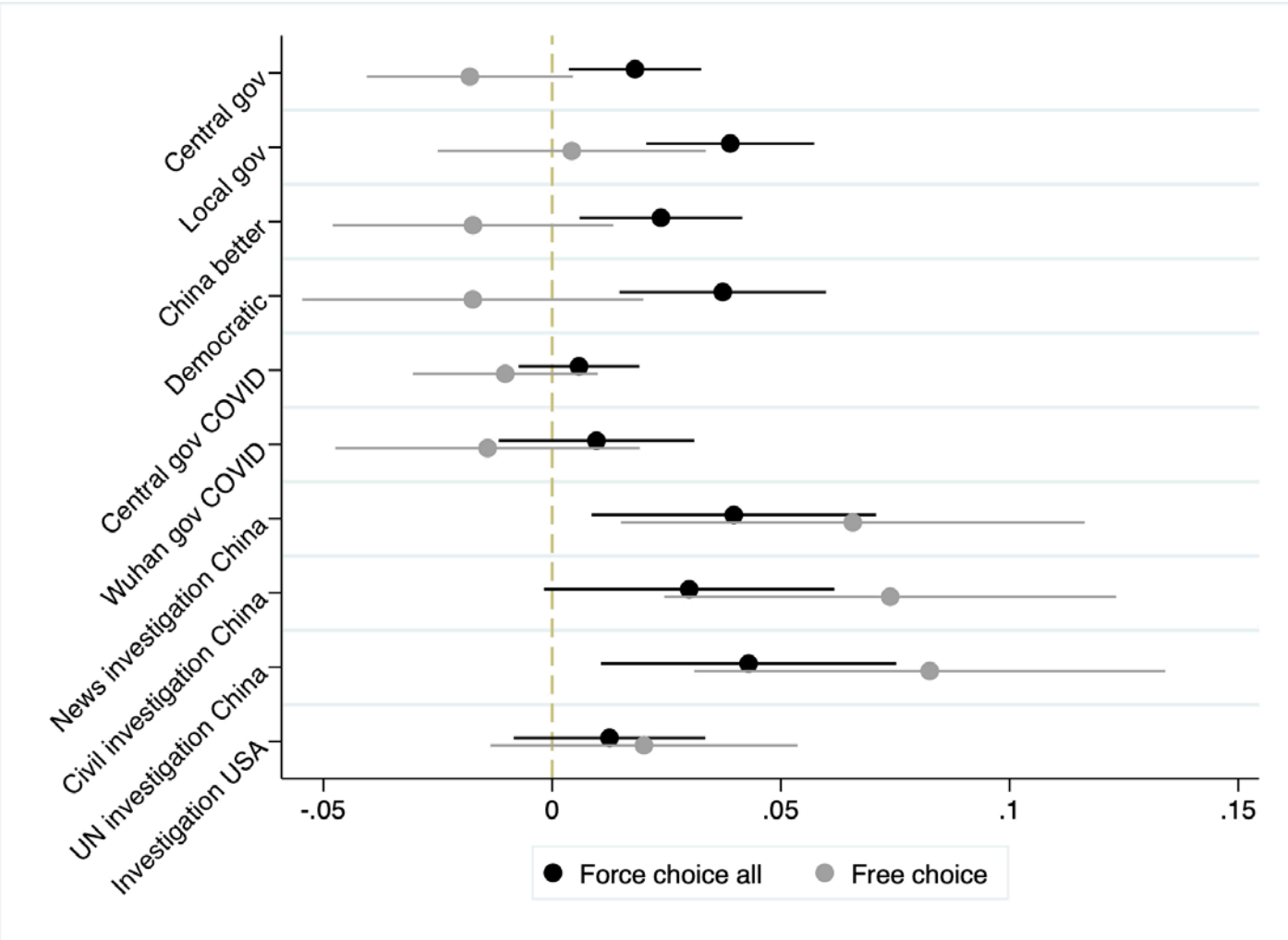

Table A6: Interactions between treatment and selective exposure. Outcomes are combined by pathway. Positive effect sizes denote more 'positive' response to questions. ***p<0.01; **p<0.05; *p<0.1

	(1)<br>Covid response	(2)<br>Country/ government	(3)<br>China inquiry
Criticism treatment	0.0153	0.0428***	0.0592***
	(0.0112)	(0.00894)	(0.0202)
Criticism choice	-0.00589	-0.0112	0.0671***
	(0.0140)	(0.0125)	(0.0250)
Treatment*Choice	-0.0245	-0.0382**	-0.0600*
	(0.0203)	(0.0177)	(0.0356)
Female	0.00961	0.00431	0.0274
	(0.00972)	(0.00826)	(0.0173)
City-province	-0.0190*	-0.00217	-0.0267
	(0.0115)	(0.00915)	(0.0195)
Age	-0.0468	-0.0173	-0.0380
	(0.0295)	(0.0245)	(0.0522)
Han	0.0246	0.0552*	-0.0389
	(0.0274)	(0.0313)	(0.0522)
Education	0.0330	0.0392	0.0222
	(0.0534)	(0.0513)	(0.0958)
Rural Hukou	-0.0150	-0.0326***	-0.0666***
	(0.0141)	(0.0108)	(0.0237)
Social use media	-0.0143	-0.00465	0.0114
	(0.0135)	(0.0119)	(0.0237)
News frequency	-0.0521*	-0.0716***	0.0235
	(0.0274)	(0.0246)	(0.0443)
International news	-0.00360	0.00487	-0.000264
	(0.00972)	(0.00816)	(0.0179)
US government attitude	-0.0147	-0.0222	0.291***
	(0.0312)	(0.0233)	(0.0534)
US people attitude	0.0271	0.0180	-0.0663
	(0.0278)	(0.0214)	(0.0510)
Ideology	0.0230	0.0392*	0.0547
	(0.0241)	(0.0211)	(0.0417)
Nationalism	0.345***	0.431***	-0.0507
	(0.0333)	(0.0301)	(0.0404)
Knowledge US politics	0.00230	-0.00590	-0.0132
	(0.0123)	(0.00959)	(0.0219)
East China	0.00750	0.0275*	0.0161
	(0.0177)	(0.0152)	(0.0289)
West China	0.00815	0.0153	0.00377
	(0.0156)	(0.0131)	(0.0232)
Constant	0.514***	0.362***	0.447***
	(0.0596)	(0.0588)	(0.0987)
Observations	1,039	1,025	1,036
R-squared	0.233	0.405	0.090

Figure A14: Interaction between choice to read criticism and criticism treatment on combined 'country' and outcome. Adjusted OLS with covariates, with 95% confidence intervals.

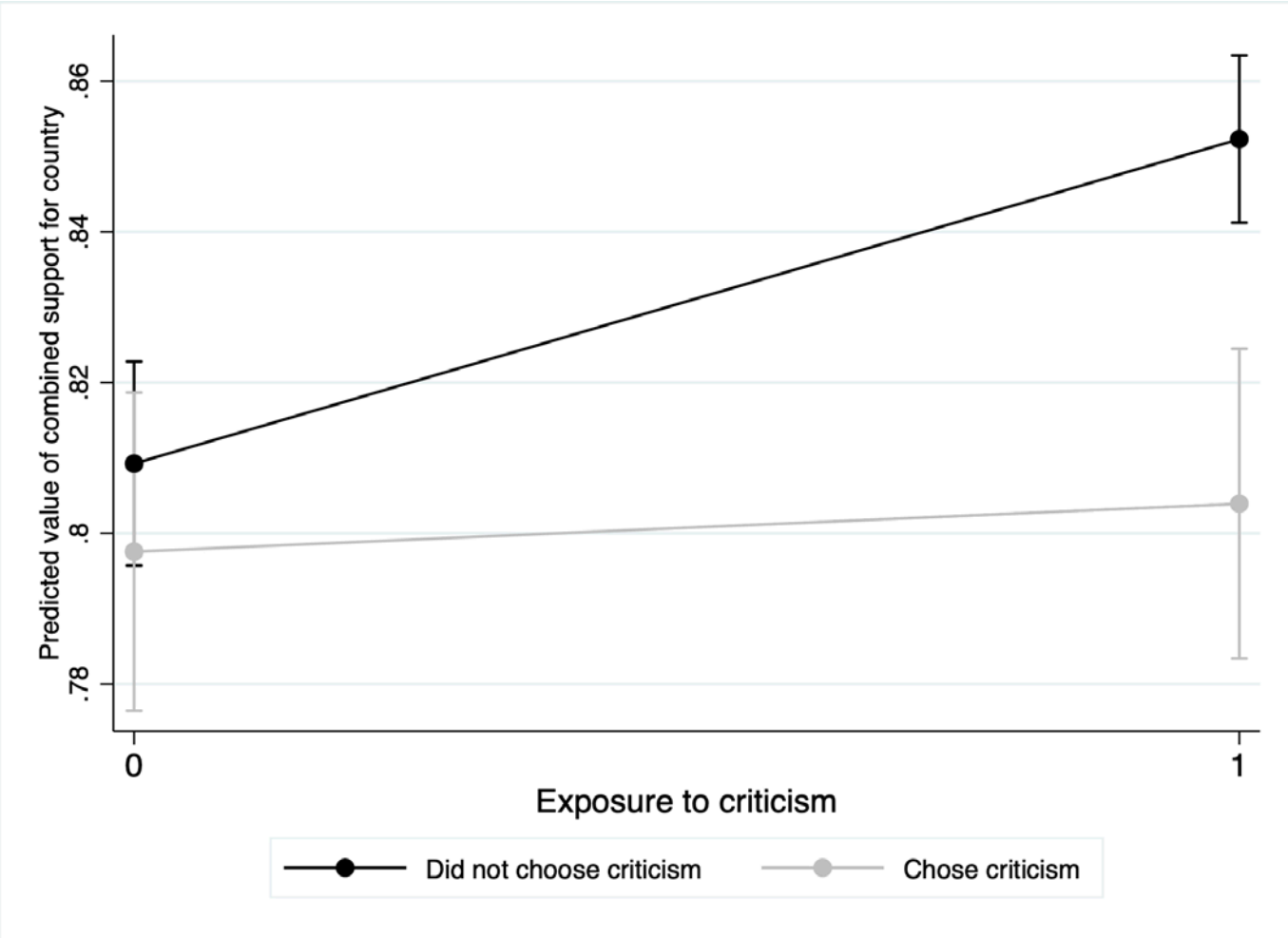

Figure A14: Interaction between choice to read criticism and criticism treatment on combined 'investigation' outcome. Adjusted OLS with covariates, with 95% confidence intervals.

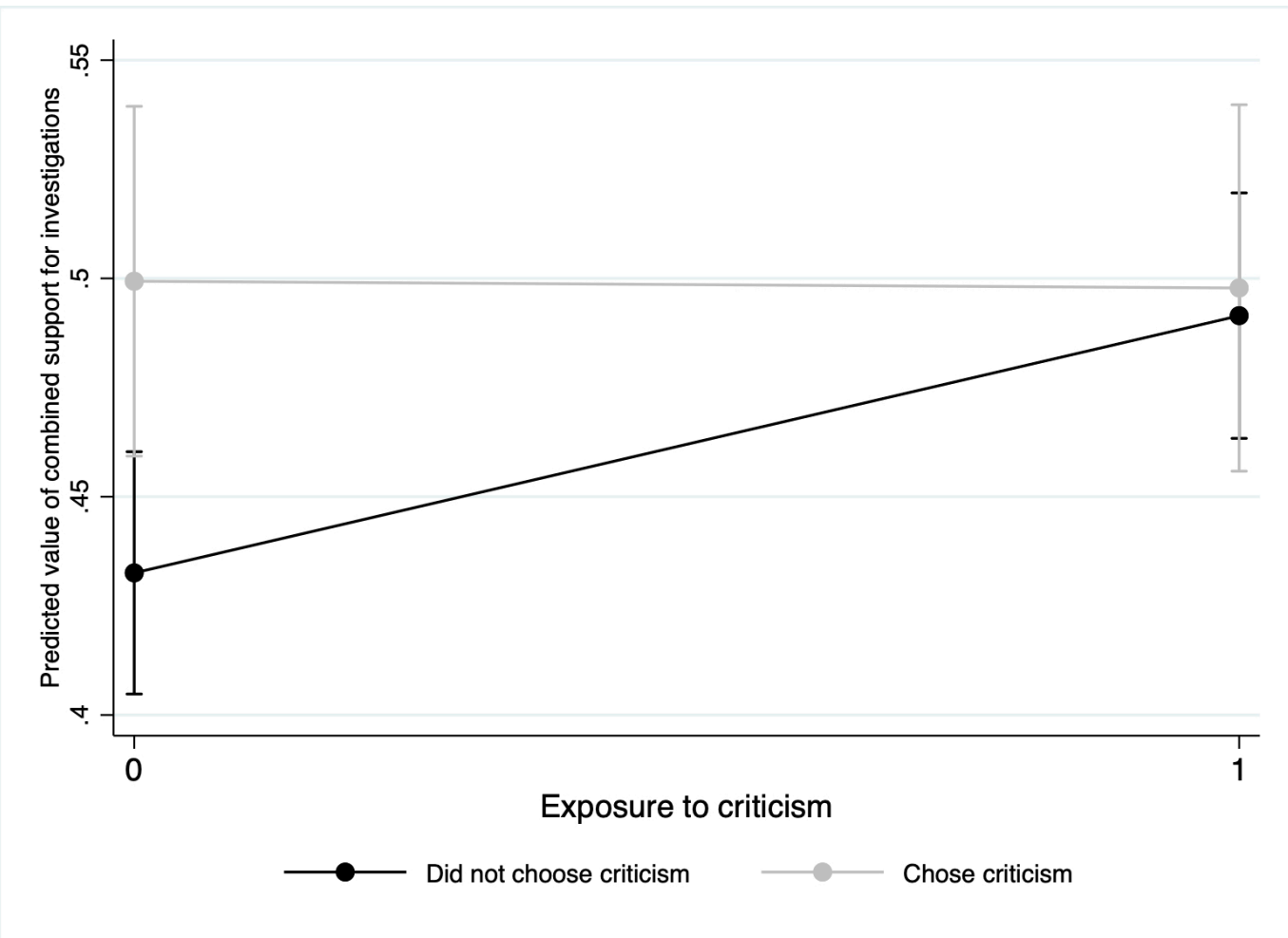

Figure A15: Results combined by pathway for respondents who indicated they would choose criticism at any point, versus those who did not indicate they would ever choose criticism.

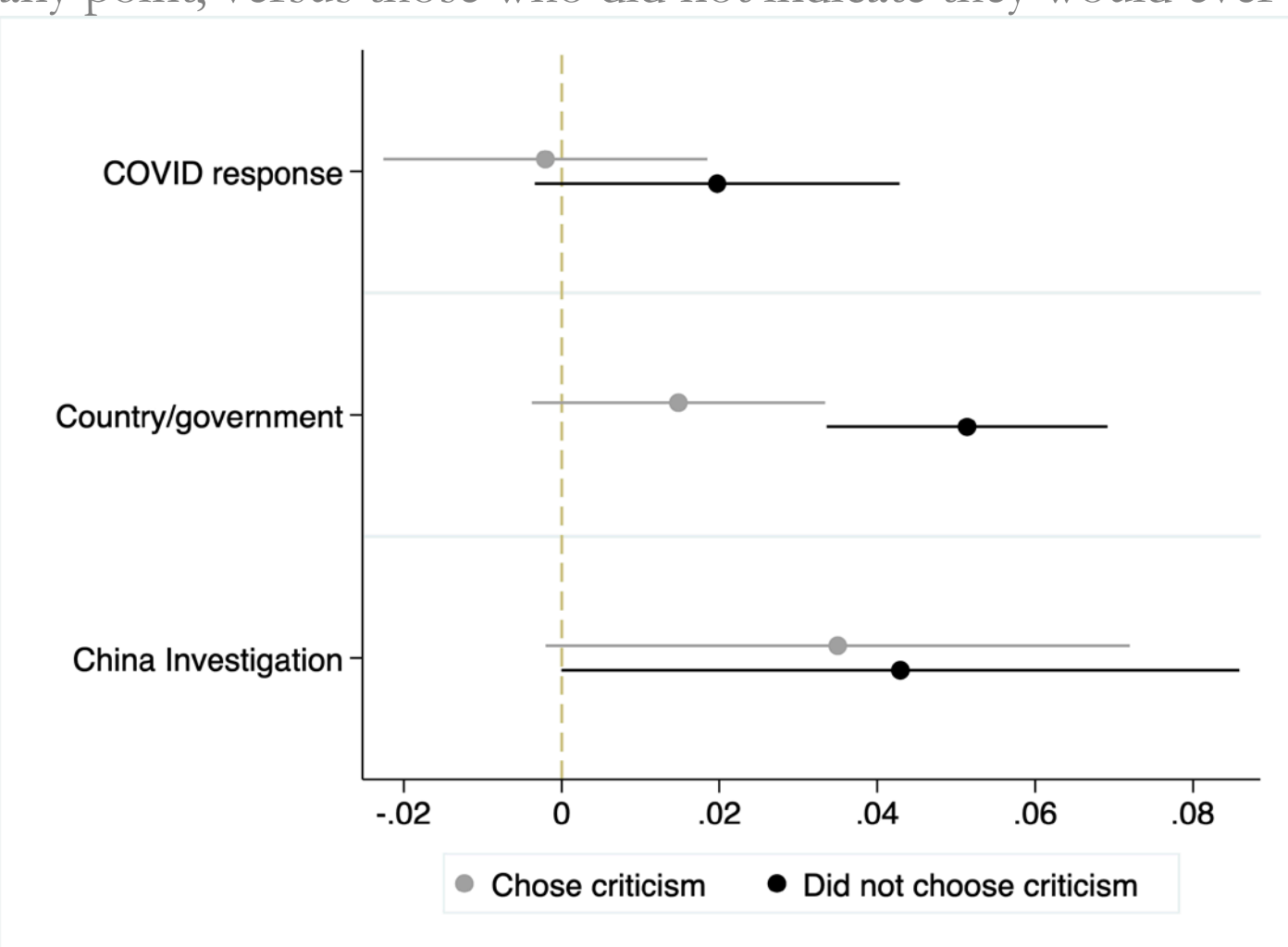

Table A7: Naive p-values from adjusted OLS regressions (from figure A5 and figure A8) alongside Anderson (2008) adjusted q-values for main variables and for combined pathways

Variable	p-value	q-value
Central government COVID	0.246	0.277
Local government COVID	0.300	0.300
Central government general	0.003	0.002
Local government general	0.0004	0.002
China better	0.014	0.021
China democratic	0.0002	0.002
Investigation UN	0.006	0.014
Investigation civil	0.044	0.057
Investigation news	0.011	0.02
Combined COVID	0.223	0.223
Combined general	0.000003	0.001
Combined investigation	0.005	0.008

V: <u>SHAMING IMPACT BROKEN DOWN BY PRETREATMENT CHARACTERISTICS</u>

For all figures unless otherwise indicated: outcomes are standardised from 0-1, using adjusted OLS with covariates. Positive effect sizes denote more 'positive' response to questions and horizontal lines represent 90% confidence intervals for estimates.

Figure A16: Impact of criticism treatment on outcomes broken down by nationalism, and by pathway. 'High' nationalism represents an answer of 'strongly agree' to the question: 'To what extent do you agree with the below sentence: "If I could choose any nationality, I would still choose to be Chinese"'?

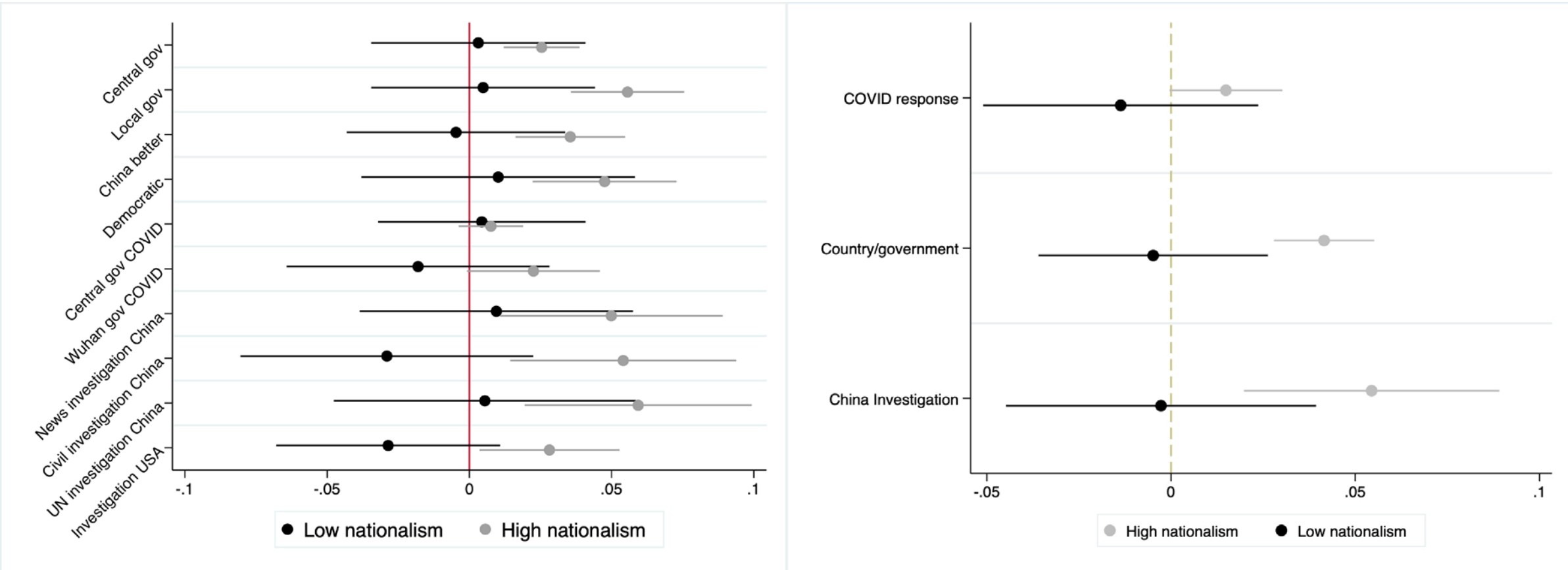

Figure A17: Under/over 35 years old

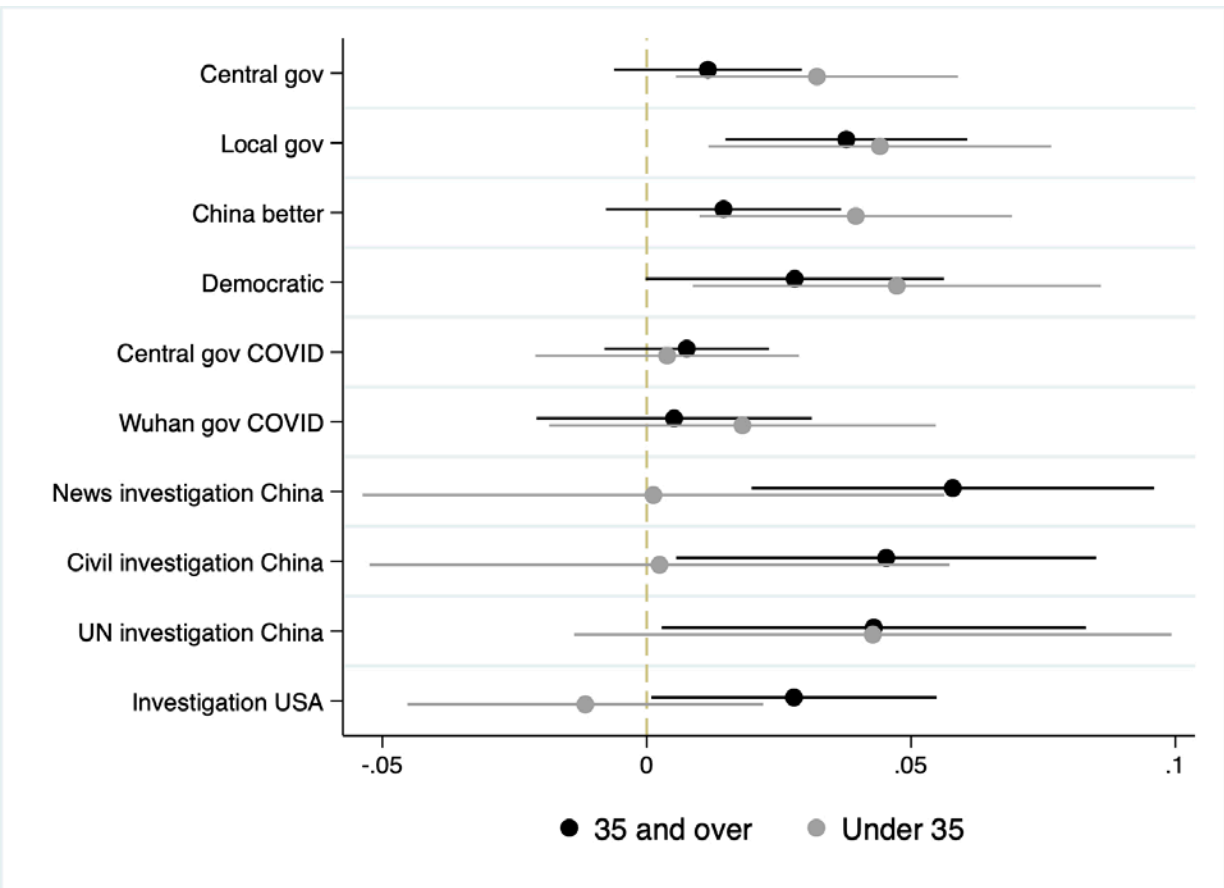

Figure A18: Urban/rural hukou

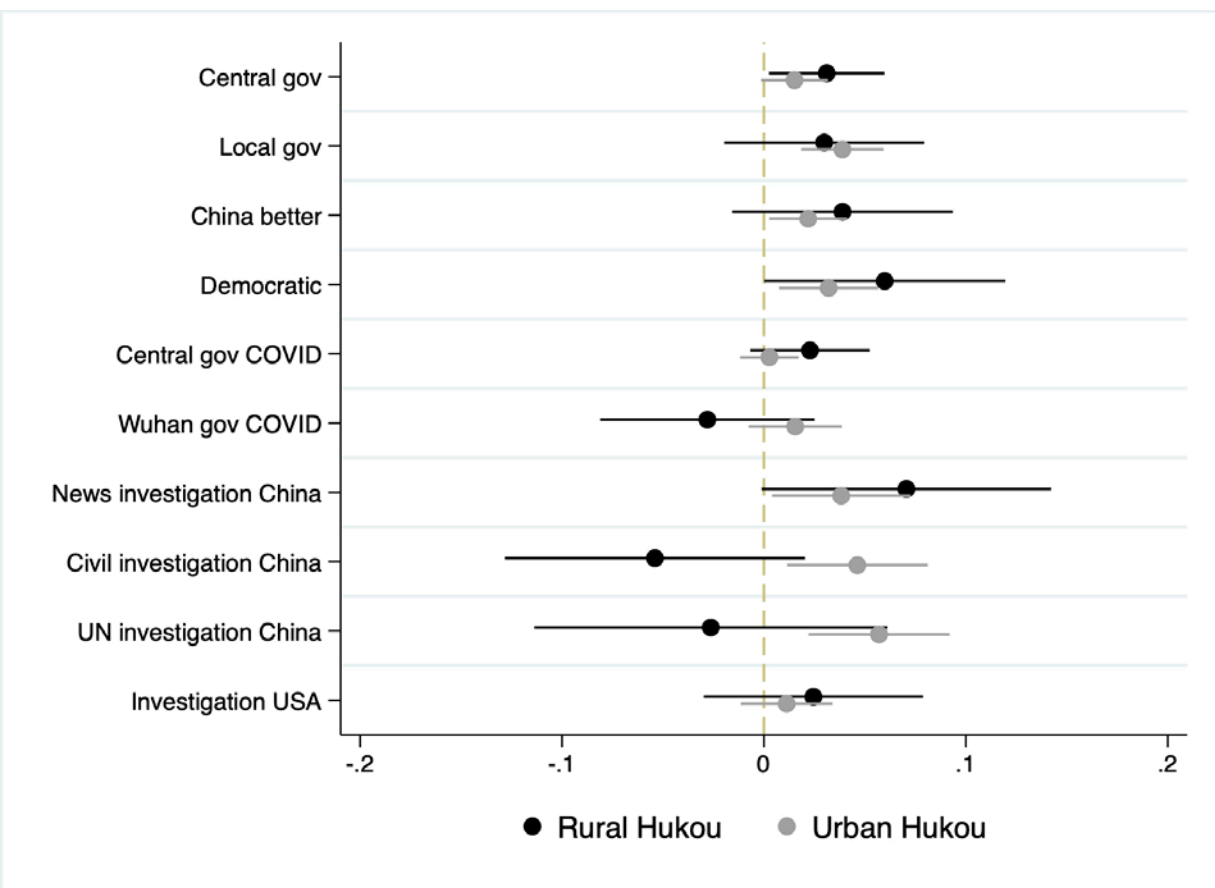

Figure A19: University/ non-university graduates

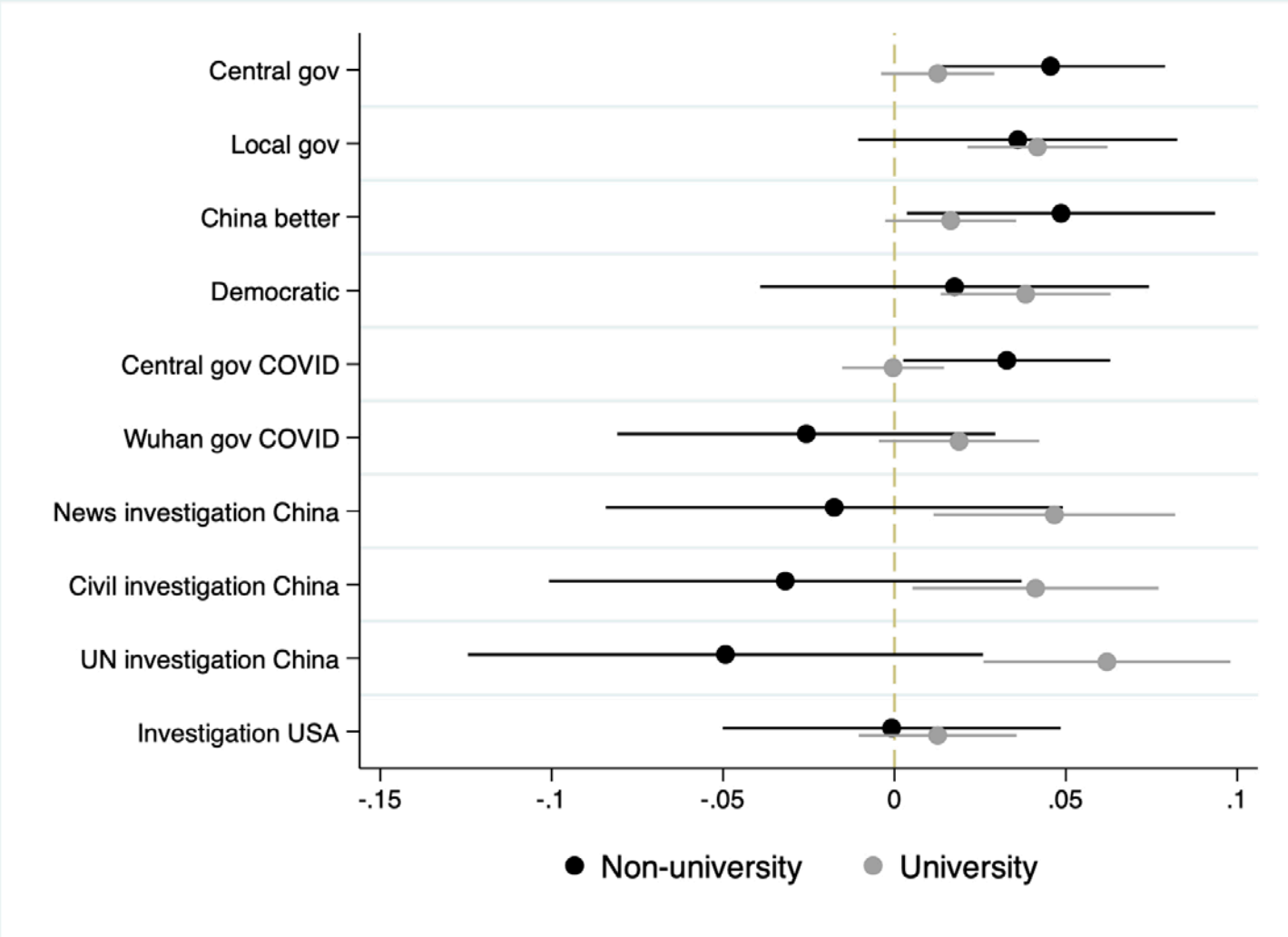

Figure A20: Male / female

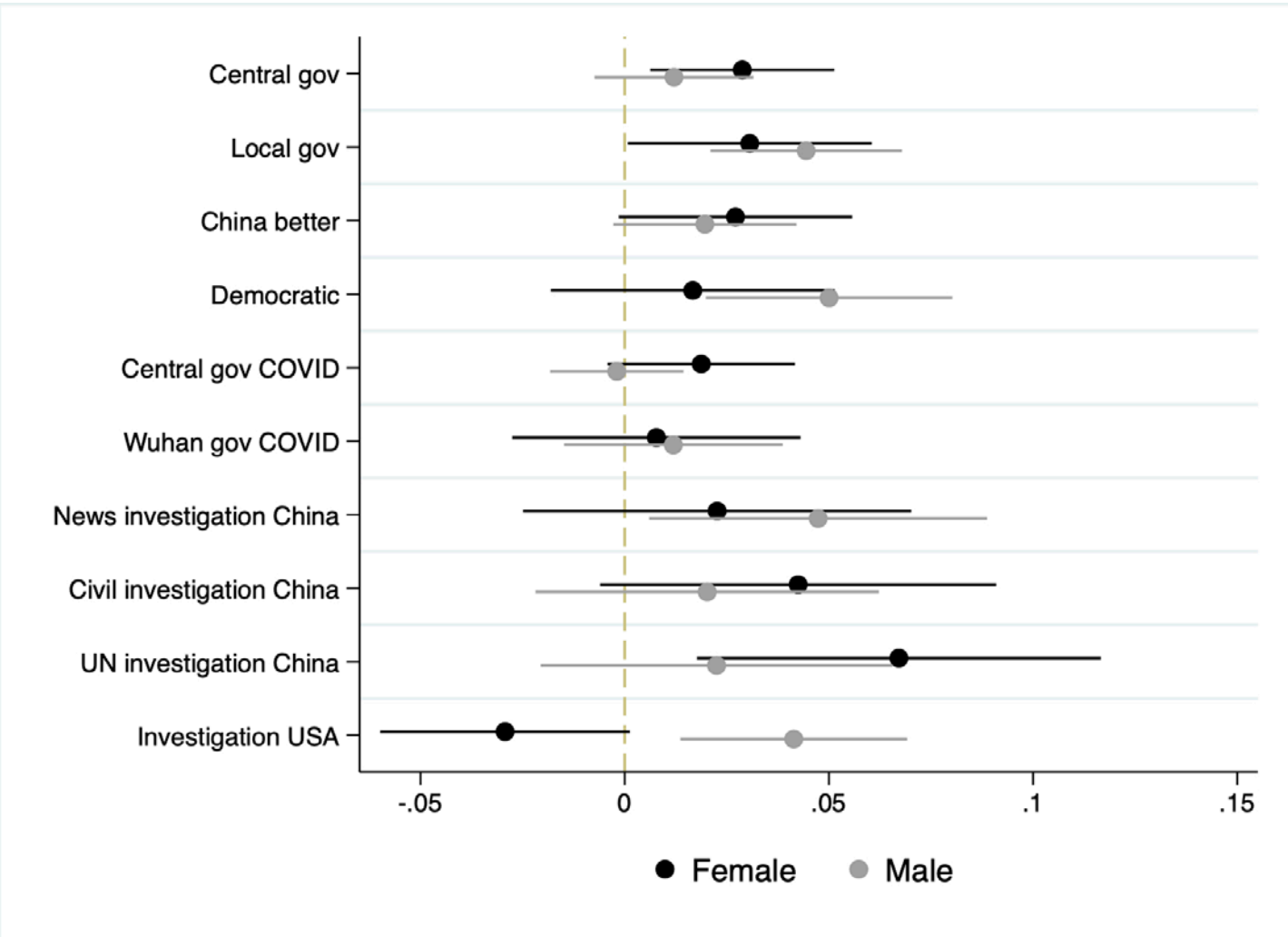

Figure A21: Like/dislike US government

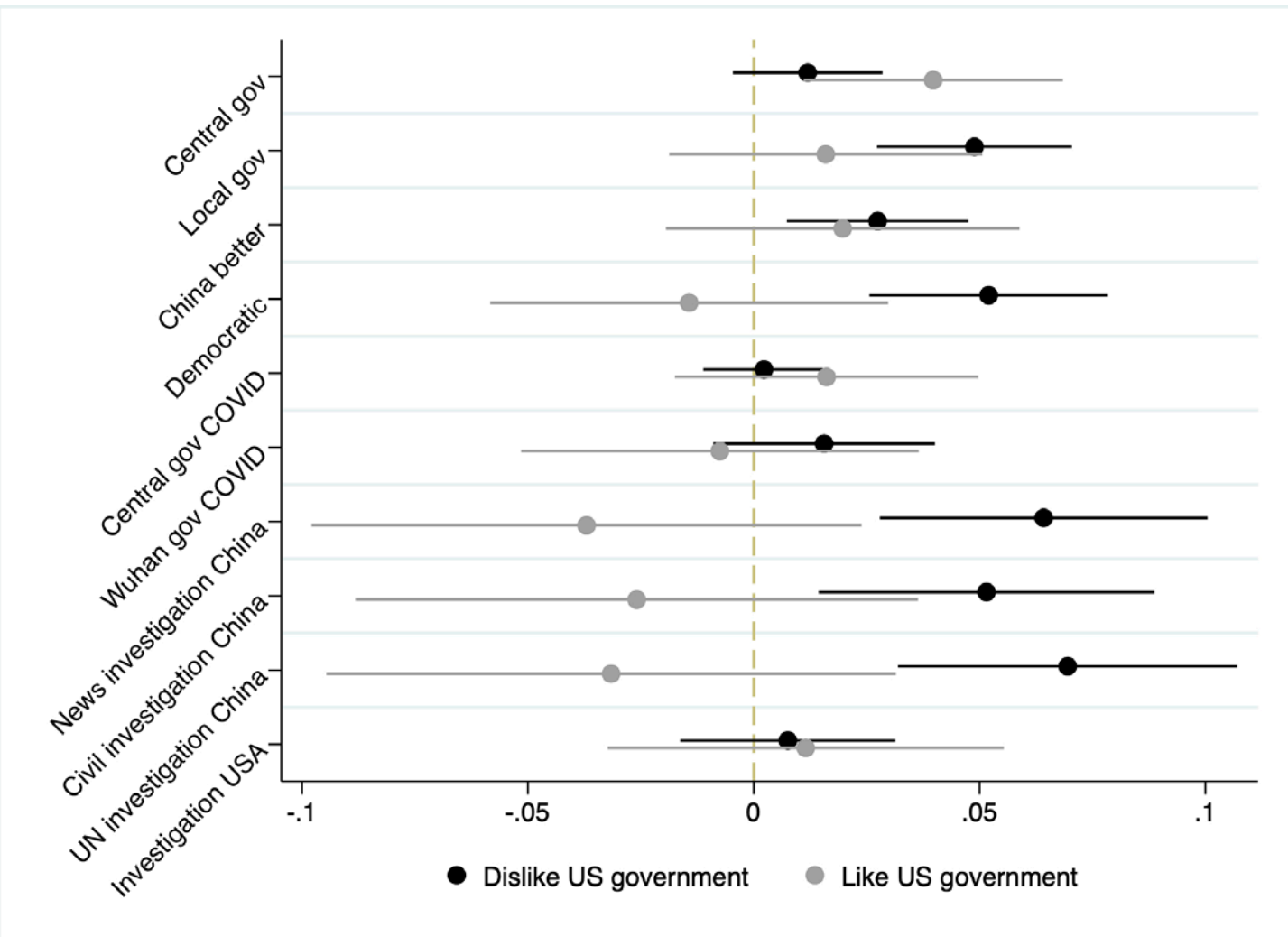

VI: Impact of Headlines on Control Group

One consequence of this design is that all respondents saw a headline referencing U.S. criticism of China's COVID-19 response, regardless of whether they were actually given that story to read (also see Huang & Yeh 2017). As discussed in the article, this means that our main results should be properly understood as measuring the difference between seeing a headline about shaming (amongst two others) plus reading a neutral article – versus seeing a headline plus reading an article about shaming. While this does have implications for how we should accurately interpret the point estimates in the experimental findings, it does not affect the overall conclusions of the article.

We can assume that people respond to the headline with the same valence as they respond to the full article (regardless of whether they chose to read it); but that any effect size will be smaller than reading the full article. In other words, if people have a backlash to a full article about shaming, then they will also have a smaller backlash to a headline about shaming: for both 'readers' and 'non-readers'. If a full article has no impact on attitudes, then a headline will also have no impact. As such, the fact that those in the control group also read the shaming headline means that the difference between control and treatment in our study is likely to be smaller than the difference between control and treatment if the control group had not seen the shaming headline.

This means that in the case when the shaming treatment leads to a backlash (i.e. general country-beliefs for non-readers), our results should, if anything, underestimate the treatment effects versus if there was no headline. If the headline does have any effects on the control group, then we would expect to see an even larger backlash if there was no headline given.

This also has implications for the null results: i.e. all the results for the 'readers'; and opinions about COVID-19 responses for the 'non-readers'. Because the control group also saw the headline about shaming, we may also be underestimating any treatment effects here. If those in the control group had not read the headline about foreign shaming, they might have given more positive (or negative) views about their government and its COVID-19 response than they did after reading the headline. So for those who chose to read the shaming, if they had not been given a headline, we cannot be sure whether the treatment would have actually led to a slight compliance or backlash for their general views about the government. And for all respondents, we cannot be sure whether the treatment would have really had no impact on their views about the government's response to COVID-19, or instead would have provoked a slight compliance or backlash.

However, the important point for this study is that any underestimations are the same for 'readers' and 'non-readers'. If headlines mean that we underestimate the level of backlash over general government support for 'readers', then headlines will do the same for 'non-readers'. For our overall conclusions about the impacts of selective exposure to be questioned, we would need the underestimation of backlash for 'readers' to be larger than the underestimation for 'non-readers'. There is nothing theoretically or empirically to suggest that could be the case. Indeed, given that 'non-readers' show a backlash after reading the article but 'readers' do not, we can assume that they would, if anything, respond to the headline in the same way, meaning that if anything, any underestimation is more likely to apply to 'non-readers'.

The study's goal is to show that people who choose to read about foreign shaming respond in a qualitatively different way to those who choose not to. The design is a simplified version of how people choose news stories in real life, so the quantitative point estimates of the level of backlash may not exactly reflect real-life responses. But the qualitative patterns of responses still hold: after being given an article about U.S. shaming, Chinese people who had originally chosen to read it are significantly less likely to rally around their government than people who had not chosen to.

VII: Literature on Naming and Shaming

Recent studies have shown that naming and shaming can substantially influence the public's support for policies towards issues from women's rights (Bush & Jamal 2015; Gruffydd-Jones 2022), criminal justice (Terman & Gruffydd-Jones 2019; Greenhill & Reiter 2021), minority rights (Kohno et al. 2023; Bassam-Nygate 2024; Abramson et al. 2024), climate change (Tingley & Tomz 2021; Koliev et al. 2022), trade policies (Gueorguiev et al. 2020), to democracy (Gruffydd-Jones 2019) and their leaders (Terman 2023).

The evidence on naming and shaming's impact on public opinion is mixed, however. Some authors find that it does indeed increase people's support for compliance with international norms and desire for improvements in their government's behaviour (Davis, Murdie, & Steinmetz 2012; Ausderan 2014; Tingley & Tomz 2021; Koliev et al. 2022). Others find that shaming may backfire, making people more supportive of their government and its policies (Gruffydd-Jones 2019; 2022; Snyder 2020; Greenhill & Reiter 2022; Terman 2023; Kohno et al. 2023; Abramson et al. 2024; Bassam-Nygate 2024).

To date there is no unified theoretical framework that explains the impact of shaming on public opinion, but from the most recent empirical studies we can draw a few core findings. Most of the studies that find shaming leads to a backlash are in places where there is a long history of international pressure over the shamed issue (in particular human rights): most notably Israel (Abramson et al. 2024; Bassam-Nygate 2024), Iran (Terman 2019), China (Gruffydd-Jones 2019; 2022), and Myanmar (Kohno et al. 2023).

These are places where the target regime has explicitly weaponised international pressure and deliberately tied it to the national identity. There is less evidence of a strong backlash in places where there has not been this history of international shaming over the issue, or no systematic government attempts to weaponise it, from Brazil and Nepal to the UK and the U.S. (Ausderan 2014; Tingley & Tomz 2021; Spektor et al. 2022; Gruffydd-Jones 2022; Terman 2023; Kohno et al. 2023). Other studies find that in places where leaders have politicised international pressure as an attack on the regime, and tied it explicitly in to their partisan political identities, foreign shaming is likely to have negative impacts amongst regime or government supporters (Bush & Jamal 2015; Kohno et al. 2023; Morisson 2023).

Human rights are especially likely to see this kind of backlash, simply because they induce by far the most shaming, and given their sensitivity, target states are far more likely to see shaming as a genuine threat. They will, therefore, be more likely to devote resources to weaponising the shaming in their domestic and international propaganda. This kind of nationalisation or politicisation is especially likely on issues that are more easily connected to national, religious, ethnic or partisan identities, like separatism (Gruffydd-Jones 2022; Abramson et al. 2024), or where the shaming can be more easily connected to a hostile geopolitical attack than genuine concern for the welfare of the target state (Snyder 2020; Gueorguiev et al. 2020; Gruffydd-Jones 2022; Terman 2023).

This study implies that selective exposure will be most likely to modify the impacts of foreign shaming in these kinds of circumstances: where shaming is viewed as an attack on the nation and so pushes more nationalist citizens to avoid it – but also to fight back if they are exposed. Studies in these places and under these conditions are the most likely to show negative ATEs in forced-choice experiments, but will only approximate those ATEs in real life when the shaming is broadcast widely. However, in these places, when the conditions are in place for shaming to evoke a backlash, but the shaming only reaches those who actively seek it out or choose to read it, the real-life impacts will be far less negative than the ATEs from experimental studies.

VIII: PRE-ANALYSIS PLAN

(Project plan found at: https://osf.io/cq7wb/overview?view_only=2c4d2d775b5d43fd8700281698d2a468 and details are copied in full below. Link to time-stamped plan will be provided if paper accepted).

1. *Study Information*

Hypotheses

The stronger Chinese citizens' national attachment, the more likely they will be to choose to read news about American criticism of their government. The more negative Chinese citizens' opinions of the United States, the more likely they will be to choose to read news about American criticism of their government. For Chinese citizens who choose to read news about American criticism of their government, the criticism will make them more positive about their government's performance. For Chinese citizens who do not choose to read news about American criticism of their government, the criticism will make them more negative about their government's performance.

2. *Design Plan*

Study type

Experiment - A researcher randomly assigns treatments to study subjects, this includes field or lab experiments. This is also known as an intervention experiment and includes randomized controlled trials.

Blinding

For studies that involve human subjects, they will not know the treatment group to which they have been assigned.

Is there any additional blinding in this study?

None

Study design

Between subjects design with one factor (Criticism) and 2 levels (control news story, and criticism news story)

No files selected

Randomization

We use block randomisation, where each participant will be randomly assigned to one of the two equally sized, predetermined blocks. The randomisation will be carried out by Qualtrics' automatic randomisation function.

3. *Sampling Plan*

Existing Data

Registration prior to creation of data
Explanation of existing data

None

Data collection procedures

The survey will be administered through a Chinese market research company's online panels. Members of the panel have chosen to join with the goal of completing online surveys for possible rewards. Possible survey respondents will receive a link in their email inviting them to take part in a survey on their social attitudes, which will be hosted on Qualtrics, an American survey website. They can choose freely whether to enter the survey or not. The participant population is representative of the Chinese online population; split evenly between men and women; only for Chinese citizens. The study will only include respondents over the age of 18.

No files selected

Sample size

Our target sample size is 1100 participants, and we will stop recruiting once this number has been reached.

Sample size rationale

Funding is only provided for a total of 1100 participants (at $2 a participant).
Stopping rule

We have full control over the exact sample size.

4. *Variables*

Manipulated variables

We manipulated whether respondents would read about foreign criticism of China's COVID-19 response, or an unrelated news story (about entertainment). There were two levels to this variable.

No files selected

Measured variables

We will measure three main variables. The non-manipulated independent variable will be respondents' news preference (whether respondents freely indicated that they would rather read a story about foreign criticism as opposed to one other news story on the same topic or one entertainment story). The first outcome variable will be respondents' attitudes towards the Chinese government's reaction to the COVID-19 outbreak in January 2020. We will measure this by asking respondents: "how would you rate the local Wuhan government's response to the January 2020 COVID outbreak?" and "how would you rate the central government's response to the January 2020 COVID outbreak?" on a 0-100 scale. The second outcome variable will be respondents' willingness to support activism over the COVID-19 outbreak in January 2020. We will measure this by asking respondents how much they agree with the following three statements (on a 5-point scale from Strongly Disagree to Strongly Agree): "The United Nations should launch an independent inquiry into the Chinese government's handling of the COVID-19 outbreak"; "Chinese newspapers should launch an independent inquiry into the Chinese government's handling of the COVID-19 outbreak"; "Chinese NGOs should launch an independent inquiry into the Chinese government's handling of the COVID-19 outbreak". They will then be asked: "If you could buy "Fang Fang's 'Wuhan Diary', how willing would you be to buy it?" on a scale from 0-100. The third outcome variable will be respondents' satisfaction with their government in general. We will measure this by asking respondents: "How satisfied are you with your local government"; and "How satisfied are you with your central government", on a 0-100 scale. To test their overall satisfaction with their country, they will also be asked how much they agree (from Strongly Disagree to Strongly Agree) with the statement "Overall, China is the best country in the world".

No files selected

Indices

We will take the mean of the two questions: "Chinese newspapers should launch an independent inquiry into the Chinese government's handling of the COVID-19 outbreak"; "Chinese NGOs should launch an independent inquiry into the Chinese government's handling of the COVID-19 outbreak", to create a single measure of "support for local action over the government's handling of the COVID-19 outbreak".

No files selected

5. *Analysis Plan*

Statistical models

We will use a simple between-group t-test for differences in means, and will present the Average Treatment Effects of American criticism, as well as the Conditional Average Treatment Effects, for the impact of American criticism dependent on whether respondents chose to read about criticism or not.

No files selected

Transformations

None

Inference criteria

We will use the standard $p<.05$ criteria for determining if the results are significantly different from those expected if the null hypothesis were correct.

Data exclusion

No awareness checks will be used. Outliers will be included. We will also note those who took less than 100 seconds to finish the survey and will exclude these in robustness checks. In the main analyses we will include all (non-missing) responses.

Missing data

If a subject chooses not to answer one of the outcome variables, they will not be included in that variable's analysis.

Exploratory analysis

We are interested in how demographic and other individual variables (age, gender, urban, education, ideology, national identity, news preferences) affect who chooses to read about foreign criticism. We will explore these relationships.

6. *Other*

Other

No response

IX: REFERENCES

Abramson, Y., Menon, A., & Gitlin, A. (2024) Whose Critique Matters? The Effects of Critic Identity and Audience on Public Opinion. *American Journal of Political Science*, 1–16.

Bassan-Nygate, L. (2024) The Micro-Foundations of Naming and Shaming: Evidence from Proposed Annexation of the West Bank. *Working Paper.*

Bush, S. S., & Jamal, A. A. (2015) Anti-Americanism, authoritarian politics, and attitudes about women's representation: Evidence from a survey experiment in Jordan. *International Studies Quarterly*, *59*(1), 34-45.

Davis, D.R., Murdie, A., & Steinmetz, C.G. (2012) Makers and Shapers: Human Rights INGOs and Public Opinion. *Human Rights Quarterly* 34(1): 199–224.

Greenhill, B., & Reiter, D. (2022) Naming and shaming, government messaging, and backlash effects: Experimental evidence from the Convention Against Torture. *Journal of Human Rights*: 1-20.

Gruffydd-Jones, J.J. (2019) Citizens and Condemnation: Strategic Uses of International Human Rights Pressure in Authoritarian States. *Comparative Political Studies,* 52: 579-612.

Gruffydd-Jones, J.J. (2022) *Hostile Forces. How the Chinese Communist Party Resists International Human Rights Pressure.* Oxford University Press.

Gueorguiev, D, McDowell, D., & Steinberg, D.A. (2020) The Impact of Economic Coercion on Public Opinion: The Case of US–China Currency Relations. *Journal of Conflict Resolution*, 64(9), 1555-1583.

Huang, H., & Yeh, Y. Y. (2019) Information from abroad: Foreign media, selective exposure and political support in China. *British Journal of Political Science*, *49*(2), 611-636.

Knox, D., Yamamoto, T., Baum, M. A., & Berinsky, A. J. (2019) Design, identification, and sensitivity analysis for patient preference trials. *Journal of the American Statistical Association*, *114*(528), 1532-1546.

Kohno, M., Montinola, G. R., & Winters, M. S. (2023). Foreign pressure and public opinion in target states. *World Development*, *169*, 106305.

Koliev, F., Page, D., & Tallberg, J. (2022) The domestic impact of international shaming: Evidence from climate change and human rights. *Public Opinion Quarterly*, 86(3), 748-761.

Morrison, K. (2024) Named and Shamed: International Advocacy and Public Support for Repressive Leaders. *Journal of Conflict Resolution*, *68*(2-3), 294-321.

Snyder, J. (2020) Backlash against Human Rights Shaming: Emotions in Group. *International Theory*, 12: 109-132.

Spektor, M, Mignozzetti, U., & Fasolin, G.N. (2022) Nationalist Backlash Against Foreign Climate Shaming. *Global Environmental Politics,* 22(1): 139-158

Tavakol, M., & Dennick, R. (2011) Making sense of Cronbach's alpha. *International journal of medical education*, *2*, 53.

Terman, R. (2023). *The geopolitics of shaming: When human rights pressure works—and when it backfires.* Princeton University Press.

Terman, R., & Gruffydd-Jones, J.J. (2019) Human Rights Shaming, Domestic Activism, and Nationalist Backlash. *Working Paper.*

Tingley, D, & Tomz, M. (2021) The effects of naming and shaming on public support for compliance with international agreements: an experimental analysis of the Paris Agreement. *International Organization*, 76(2): 1-24.
